# Investigation of the drug release modifying property of *Pentadesma butyracea* gum in diclofenac sodium matrix tablets

**DOI:** 10.1371/journal.pone.0341668

**Published:** 2026-01-29

**Authors:** Mary-Ann Archer, Kwabena Ofori-Kwakye, Raphael Johnson, Isaac Yaw Attah, Frederick William Akuffo Owusu, Prince George Jnr Acquah, Samuel Lugrie Kipo

**Affiliations:** 1 Department of Pharmaceutics, School of Pharmacy and Pharmaceutical Sciences, University of Cape Coast, Cape Coast, Ghana; 2 Department of Pharmaceutics, Faculty of Pharmacy and Pharmaceutical Sciences, Kwame Nkrumah University of Science and Technology, Kumasi, Ghana; 3 Department of Pharmaceutical Chemistry, School of Pharmacy and Pharmaceutical Sciences, University of Cape Coast, Cape Coast, Ghana; National University of Rosario, ARGENTINA

## Abstract

**Background:**

Natural gums have long served as essential excipients in pharmaceutical formulations, traditionally functioning as binders, disintegrants, suspending agents, and emulsifiers. Their role has expanded to include drug delivery carriers in advanced systems such as matrix formulations, gene therapy, and nanomedicine. Increasing attention has been given to natural gums over synthetic alternatives due to their non-toxicity, biodegradability, affordability, and availability. This study investigated the potential of *Pentadesma butyracea* gum as a matrix-forming agent for the extended release of diclofenac sodium.

**Methods:**

The crude gum was harvested from the stem bark of *Pentadesma butyracea* and purified using ethanol precipitation. Fourier-transform infrared spectroscopy (FTIR) was employed to assess potential interactions between the gum, diclofenac sodium, and other excipients. Matrix tablets (PM01–PM05) containing varying concentrations (15–35% ^w^/_w_) of the purified gum were prepared via wet granulation, along with a control batch (PM06) lacking the gum. Additionally, the PM01 formulation was optimised by incorporating xanthan gum at concentrations of 10–20%^w^/_w_ (PM01A–PM01C). All formulations were assessed for weight and dimensional uniformity, mechanical integrity, drug content, and *in vitro* release characteristics. Comparative dissolution profiles were assessed against an innovator brand using the similarity (f₂) and difference (f₁) factors. Drug release kinetics and mechanisms were analysed using appropriate mathematical models.

**Results:**

All batches passed the uniformity of weight and dimensions tests, drug content (99.11–101.87%), friability (0.25–0.79%), hardness (7.74–8.83 Kgf), and tensile strength (1.29–1.49 MPa). The control batch (PM06) failed to meet dissolution criteria, releasing only 60.46% of diclofenac sodium within 45 minutes. In contrast, formulations containing *P. butyracea* gum exhibited a gradual, concentration-dependent, time-mediated release, with all test batches (PM01–PM05) releasing over 50% of the drug within 45 minutes. Among the optimised formulations, PM01C exhibited a modified release profile, with 19.90 ± 0.508% of the drug released at 2 hours and 100.26 ± 2.118% at 24 hours, consistent with the British Pharmacopoeia standards. The release profile of PM01C was comparable to the reference product (f₂ = 79.35; f₁ = 2.93) and followed zero-order kinetics. PM01A and PM01B exhibited Korsmeyer-Peppas release kinetics. Mechanistically, drug release occurred via non-Fickian diffusion for PM01C and Fickian diffusion for PM01A and PM01B.

**Conclusion:**

A matrix system comprising 15%^w^/_w_ *P. butyracea* gum and ≥ 20%^w^/_w_ xanthan gum effectively extended the release of diclofenac sodium over 24 hours, demonstrating its potential application in extended-release formulations.

## 1.0. Introduction

The oral route remains the most widely preferred mode of drug administration, owing to its non-invasive nature, improved patient compliance, and convenience. It also offers greater formulation flexibility compared to other routes of administration [1,2]. Oral dosage forms are cost-effective and easily amenable to scale-up, making them suitable for both small-scale and industrial manufacturing. Globally, approximately 90% of pharmaceutical products intended for human use are administered orally, and about 84% of the top-selling drugs are delivered via this route, contributing to a market value of approximately $35 billion [2,3].

Matrix formulations are particularly advantageous over conventional immediate-release systems due to their ability to maintain therapeutic drug concentrations over extended periods. By avoiding peak plasma concentrations, they help to reduce toxicity, enhance therapeutic efficacy, and minimise drug accumulation, especially during chronic dosing. Furthermore, reduced dosing frequency associated with these systems improves patient adherence [4].

Several terminologies are used to describe oral formulations with modified drug release characteristics, such as sustained release*,* extended release*,* controlled release*,* modified release*,* and delayed release*.* Matrix tablets, a common form of modified-release dosage forms, are designed to continuously release drug over a prolonged period following a single dose administration. These are typically formulated by incorporating release-modifying polymers, which may be blended with active pharmaceutical ingredients and excipients, followed by either direct compression or granulation before compression.

Among the various types of polymers used in matrix systems, hydrophilic polymers are widely employed due to their cost-effectiveness and capability to provide desirable release profiles [5]. In hydrophilic matrix systems, the polymer undergoes swelling upon contact with aqueous fluids, forming a gel layer through which the drug is released via dissolution, erosion and/or diffusion mechanisms [1]. Common examples of such polymers include hydroxypropylmethylcellulose (HPMC), hydroxypropylcellulose (HPC), xanthan gum, and sodium alginate. In this study, the drug release-controlling potential of purified *Pentadesma butyracea* gum, a naturally derived hydrophilic polymer, was investigated as a candidate excipient for use in extended-release oral diclofenac sodium matrix tablet formulations.

## 2.0. Materials and methods

### 2.1. Materials

Diclofenac sodium (ALB Technology Ltd., USA), Trifluoroacetic acid (Oakwood Products Inc., USA), acetonitrile (PerkinElmer Inc., USA), methanol (Sankyo Chemical Co. Ltd, Japan), purified *Pentadesma butyracea* gum (7º 43’ 8.7”N, 1º 40’ 43.9”W, Nkoranza North, Ghana), talc (Multi Mineral Industries, Jodhpur), magnesium stearate (Anhui Sunhere Pharmaceutical Excipient Co. Ltd., China), phosphoric acid (Innophos Inc., USA), sodium dihydrogen orthophosphoric acid (EURCHEMnet, Italy), xanthan gum (Guangzhou ZIO Chemical Co. Ltd., China), Microcrystalline cellulose (MCC) (Shanghai Yuanri International Trade Co. Ltd., China), ethanol was obtained from the chemical store of the department of Pharmaceutics, UCC.

### 2.2. Preparation of diclofenac sodium granules using *Pentadesma butyracea* gum as matrix agent

Purified *Pentadesma butyracea* gum was incorporated into tablet formulations at concentrations ranging from 15 to 35%^w^/_w_ (batches PM01–PM05), based on the total tablet weight, as presented in Table 1. A total of five batches were developed, each with a distinct gum concentration. In preparing the granules, only essential excipients were included, namely, a diluent, a lubricant (added before compression), the active pharmaceutical ingredient (diclofenac sodium), and the release modifiers. These components were blended using the doubling-up method to specifically assess the effect of the gum as a matrix-forming agent. Granulation was accomplished using the wet granulation technique, with distilled water as the granulating fluid. The wet mass was passed through a 2360 µm sieve and then dried at 60°C for 1 hour. After cooling in a desiccator, the dried granules were re-screened using a 1190 µm sieve. The granules were subsequently stored in ziplock bags for further analyses and tablet compression. Additionally, a control batch (PM06) was formulated without the gum to assess the specific impact of *P. butyracea* gum on matrix formation. Furthermore, optimised formulations containing xanthan gum at concentrations of 10–20%^w^/_w_ (PM01A–PM01C) were developed for comparative analysis.

**Table 1:** Formula for matrix formulations.

Ingredients		Quantity per tablet (mg)								
		PM01	PM02	PM03	PM04	PM05	PM06	PM01A	PM01B	PM01C
Diclofenac sodium (API)		100	100	100	100	100	100	100	100	100
Release modifier	*Pentadesma butyracea* gum	60	80	100	120	140	–	60	60	60
	Xanthan gum	–	–	–	–	–	–	40	60	80
MCC		232	212	192	172	152	292	192	172	152
Magnesium stearate		4	4	4	4	4	4	4	4	4
Talc		4	4	4	4	4	4	4	4	4

### 2.3. Fourier transform infrared (FTIR) analysis for compatibility studies

Approximately 5 mg of each sample was placed in the sample holder of the FTIR spectrophotometer (Bruker), and their respective spectra were recorded. The resulting spectra were superimposed and compared against the reference library to assess spectral compatibility [6,7].

### 2.4. Evaluation of the flow properties of the diclofenac sodium granules before compression

#### 2.4.1. Assessment of the bulk and tapped densities, Hausner ratio and % compressibility of the diclofenac sodium granules.

A 10 g quantity of formulated granules was accurately weighed and transferred through a funnel into a 100 mL graduated cylinder. The cylinder was gently tapped twice to dislodge any adhering powder from the inner walls. The initial volume (V_b_) was recorded as the bulk volume.

Subsequently, the cylinder was tapped 50 times from a height of 2.5 cm onto a wooden surface to allow the powder to settle to a constant volume, noted as the final volume (V_t_).

The bulk (or apparent) density (D_b_) for each batch of granules was calculated using the equation [8]:


$${\text{D}}_{\text{b}}\;=\;\text{m}\text{a}\text{s}\text{s}\;/\;{\text{V}}_{\text{b}}$$ (1)

The tapped density (D_t_) for each batch of granules was calculated using [8]:


$${\text{D}}_{\text{t}}\;=\;\text{m}\text{a}\text{s}\text{s}\;/\;{\text{V}}_{\text{t}}$$ (2)

$$\;\text{H}\text{a}\text{u}\text{s}\text{n}\text{e}\text{r}’\text{s}\;\text{r}\text{a}\text{t}\text{i}\text{o}\;=\;{\text{D}}_{\text{t}}\;/\;{\text{D}}_{\text{b}}$$ (3)

$$\%\;\text{c}\text{o}\text{m}\text{p}\text{r}\text{e}\text{s}\text{s}\text{i}\text{b}\text{i}\text{l}\text{i}\text{t}\text{y}\;=\;[({\text{D}}_{\text{t}}\;–\;{\text{D}}_{\text{b}})\;/\;{\text{D}}_{\text{t}}]\;\times \;\text{1}\text{0}\text{0}$$ (4)

#### 2.4.2. Evaluation of the angle of repose of the diclofenac sodium granules.

The angle of repose, a critical indicator of powder flowability, was determined using the fixed height method. For each batch of granules, the sample was allowed to flow freely through a funnel fixed at a vertical distance of 2 cm from the horizontal surface. This facilitated the formation of a conical heap. The base of the cone was marked, and the granules were removed to measure the diameter of the base. The average diameter was calculated from two perpendicular measurements, and the radius (r) was determined accordingly. The height (h) of the cone was also measured. The angle of repose (θ) was then computed using the equation [8]:


$$\begin{array}{c} \theta \;=\;tan-¹(h/r) \end{array}$$ (5)

### 2.5. The compression of the diclofenac sodium granules into tablets

**Fig 1:**
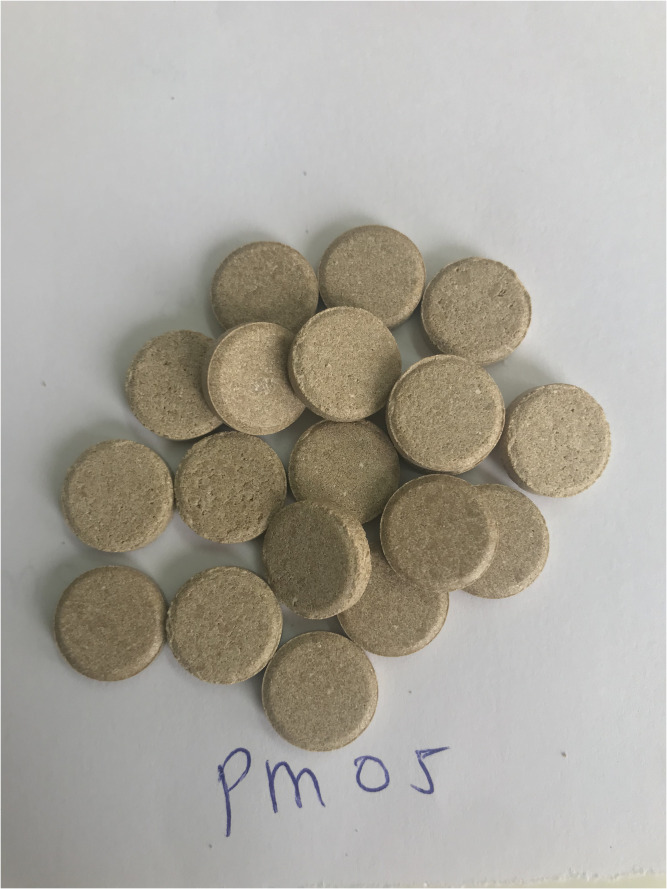
Image of a sample of diclofenac sodium matrix tablets.

The diclofenac sodium granules for each formulation were compressed into tablets (Fig 1) using a Heysu ZP35D rotary tablet compression machine (Shanghai, China), after lubricating with the respective amounts of talc and magnesium stearate.

### 2.6. Pharmaceutical quality evaluation of compressed diclofenac sodium tablets (PM01-PM06; PM01A-PM01C)

#### 2.6.1. Determination of the thickness and diameter of PM01-PM06, and PM01A-PM01C.

The diameter and thickness of ten tablets from each formulation batch were measured using a digital Vernier caliper (SKU: DVC-W-150, China). The mean values and standard deviations were subsequently calculated to assess uniformity in tablet dimensions, which is essential for ensuring consistent dosage and mechanical strength [8,9].

#### 2.6.2. Uniformity of weight test of PM01-PM06 and PM01A-PM01C.

Twenty randomly selected tablets from each formulation batch were collectively weighed to determine the mean tablet weight. Subsequently, each tablet was weighed individually, and the percentage deviation from the mean weight was calculated to evaluate weight uniformity, as recommended by pharmacopoeial guidelines [6].

#### 2.6.3. Friability test of PM01-PM06 and PM01A-PM01C.

To assess the mechanical integrity of the formulations, tablets from each formulation batch, with a total weight equivalent to 6.5 g, were individually dusted and placed in a friabilator (Panmex Laboratories and Industrial Motors, Model 902, Delhi). The apparatus was operated at 25 rpm for 100 revolutions (equivalent to 4 minutes), allowing the tablets to undergo mechanical tumbling within the rotating drum. After the test, the tablets were removed, excluding any broken fragments, dusted, and reweighed. The friability was calculated as the percentage weight loss using the difference between the initial and final tablet weights [6].

#### 2.6.4. Evaluation of tablet hardness and tensile strength of PM01-PM06 and PM01A-PM01C.

The mechanical strength and resistance of each formulation batch to crushing were evaluated by randomly selecting 5 tablets from each batch. Each tablet was individually positioned between the spindle and anvil of a DKB hardness tester (Serial No. 123), with the calibration scale adjusted to zero before measurement. A compressive force was gradually applied until the tablet fractured, and the breaking point was recorded in kilogram-force (kgf) [6,8].

The tensile strength was calculated by [6]:


Tensile  strength = 2F / (πDt), (6)

where F is tablet hardness, t is tablet thickness, and D is the tablet diameter

#### 2.6.5. Assay of diclofenac sodium formulation batches by HPLC.

For each formulation batch, twenty [20] tablets were randomly selected, weighed, and triturated into a fine powder. A quantity of the powdered sample, equivalent to 0.5 g of diclofenac sodium, was accurately weighed and transferred into a volumetric vessel containing 800 mL of methanol. The mixture was subjected to sonication to ensure complete dissolution. The resulting solution was then diluted with the mobile phase to achieve a final diclofenac sodium concentration of 0.005%^w^/_v_. The diclofenac sodium content in each formulation batch was quantified using a British Pharmacopoeia (2022) validated high-performance liquid chromatography (HPLC) method, with the chromatographic conditions as shown in Table 2 [6].

**Table 2:** Chromatographic conditions applied in the assay of formulation batches PM01-PM06 and PM01A-PM01C.

Parameter	Conditions
Chromatograph	Agilent HPLC (1260)
Stationary Phase	25 cm x 4.6 mm; 5 µm, Zorbex C8
Mobile phase	Methanol: 0.1% Phosphoric acid and 0.16% sodium dihydrogen orthophosphate (80: 20)
Detection wavelength	254 nm
Elution method	Isocratic
Temperature	Ambient
Flow rate	1 mL/min
Run time	7 minutes
Injection volume	20 µL

#### 2.6.6. *In-vitro* release studies of diclofenac sodium from matrix tablets.

***HPLC method development for quantifying in-vitro diclofenac release from formulations PM01-PM06 and PM01A-PM01C.* Preparation of stock solution**. An accurately weighed 0.30 g of diclofenac sodium was placed into a clean glass beaker. The drug was dissolved in 300 mL of phosphate buffer (pH 6.8) with sonication. The obtained solution was transferred into a 500 mL volumetric flask and diluted to volume with the same phosphate buffer to yield a 0.06%^w^/_v_ solution. After stoppering, the flask was shaken thoroughly to ensure homogeneity. This stock solution served as the source for preparing test solutions at various analyte concentrations by appropriate dilution with phosphate buffer (pH 6.8) for use in High-Performance Liquid Chromatography (HPLC) method development.

**HPLC analytical method development.** The method was developed with consideration for the ability to detect and quantify diclofenac sodium, cost-effectiveness, reagent availability, and analysis time. The HPLC chromatographic conditions were progressively optimised through iterative adjustments until optimal separation was achieved, characterised by a distinct peak for diclofenac sodium (Table 3).

**Table 3:** Chromatographic conditions for the developed method.

Parameter	Conditions
Chromatograph	PerkinElmer Flexar
Stationary Phase	C18 phenomenex (150 x 4.6 mm; 5 µm)
Mobile phase	0.1% ^v^/_v_ Trifluoroacetic acid: acetonitrile (25%: 75%)
Detection wavelength	254 nm
Elution method	Isocratic
Temperature	25 ºC
Flow rate	1 mL/min
Run time	5 minutes
Injection volume	20 µL

**Establishment of chromatographic method parameters.** *Determination of the appropriate stationary phase.* One of the fundamental principles in HPLC method development is that the polarity of the analyte should be opposite to that of the stationary phase. This ensures adequate interaction between the analyte and the column. Thus, the stationary phase should be strong enough to retain the compound, yet weak enough to allow elution within a defined run time. For this study, a run time of approximately 5 minutes was targeted. Given that the diclofenac sodium under investigation exhibits intermediate polarity, possessing both polar and non-polar characteristics, a C18 (150 mm × 4.6 mm, 5 µm particle size) column (Table 3) was chosen due to its longer alkyl chain, which enhances hydrophobic interactions and provides improved retention characteristics for moderately non-polar compounds [10,11].

*Selection of an appropriate mobile phase***.** The mixture of 0.1% ^w^/_v_ Trifluoroacetic acid and acetonitrile in the ratio of 25: 75 (Table 3) was chosen because this mobile phase composition is relatively nonpolar, making it suitable for eluting moderately to strongly nonpolar compounds or reducing retention time for compounds with intermediate polarity like diclofenac sodium [10,12].

*Selection of wavelength and injection volume for the analysis.* With guidance from the British Pharmacopoeia [6], the injection volume and wavelength chosen were 20 µL and 254 nm, respectively (Table 3).

**Assessment of the Specificity of the HPLC developed method.** The working solutions of the diclofenac sodium in matrix tablet (0.02% ^w^/_v_) and the conventional tablet (0.4% ^w^/_v_) were prepared and analysed using the developed HPLC method. The blank was also subjected to the same chromatographic conditions. The chromatograms of the analytes obtained from multiple determinations were subsequently compared to evaluate the method’s performance.

***Investigation of the Linearity of the HPLC developed method*.** Standard calibration curve, expressed as a linear regression plot, was generated for diclofenac sodium using concentrations 0.03% ^w^/_v,_ 0.015% ^w^/_v_, 0.0075% ^w^/_v_, 0.00375% ^w^/_v_, 0.001875% ^w^/_v_ and 0.0009375% ^w^/_v,_ which were prepared via serial dilution of the stock. Each concentration was analysed in triplicate runs, and the corresponding peak areas were recorded. A plot of the peak area versus concentration was then constructed to determine the coefficient of determination (R²), assessing linearity.

**Evaluation of the Limit of Detection (LOQ) and the Limit of Quantification (LOQ) of the HPLC developed method.** According to the guidelines set by the International Council for Harmonisation of Technical Requirements for Pharmaceuticals for Human Use (ICH) [13], the limit of detection and the limit of quantification for analytical methods should be calculated using the following equations [13]:


$$\text{LOD}\;=\frac{\text{3}.\text{3}\sigma }{S}$$ (7)

$$\text{LOQ}\;=\frac{\text{1}\text{0}\sigma }{S}$$ (8)

where *S* represents the slope of the calibration curve and *σ* denotes the standard deviation of the response [13].

**Assessment of the accuracy of the HPLC developed method.** The accuracy of the developed method was evaluated by analysing three concentration levels for diclofenac sodium (0.0075% ^w^/_v_, 0.015% ^w^/_v_ and 0.03% ^w^/_v_), corresponding to 50%, 100% and 150% of its respective working concentrations. Each concentration was prepared in triplicate and analysed using the developed HPLC method. The average percentage recovery was then calculated for each level to assess the method’s accuracy

**Assessment of the precision of the HPLC developed method.** Repeatability of the analytical method was assessed by analysing a 0.015% ^w^/_v_ diclofenac sodium solution six consecutive times, and calculating the mean percentage recovery and relative standard deviation (RSD). The RSD was determined using the formula [13]:


$$\text{RSD}\;(\%)\;=\;(\sigma \;/\;\text{x}\bar{})\;\times \;\text{100}$$ (9)

where *σ* represents the standard deviation and *x̄* is the mean of the peak area values.

To evaluate the intraday precision, the same test samples were reanalysed after six hours under identical conditions, and the corresponding RSD was calculated. To assess the intermediate precision, the same working diclofenac sodium solution was analysed six times, each day for three consecutive days. The mean percentage recovery and the RSD were subsequently evaluated for each day.

**Assessment of the robustness of the HPLC developed method.** The robustness of the developed HPLC method was evaluated by introducing deliberate variations to key analytical parameters. Specifically, the column source was changed to a Microsorb-MV 100−5 C18 (150 × 4.6 mm), the HPLC instrument was substituted with an alternative model (Agilent 1260 infintiy II), and the mobile phase was modified to consist of 80 parts methanol and 20 parts buffer (pH 2.5). The buffer was prepared by mixing equal volumes of 0.1%^w^/_w_ phosphoric acid and 0.16%^w^/_v_ sodium dihydrogen orthophosphate. Diclofenac sodium solutions at concentrations of 0.003% ^w^/_v_, 0.0075% ^w^/_v_, and 0.015% ^w^/_v_ were analysed, each in triplicate. The mean percentage recoveries were calculated for each concentration, and the relative standard deviations (RSDs) were determined to assess the consistency of the method under the altered conditions.

***Procedure for dissolution studies*.** The *in-vitro* dissolution behaviour of the matrix tablets formulated exclusively with *Pentadesma butyracea* gum (PM01–PM05) was evaluated using the United States Pharmacopoeia (USP) Dissolution Apparatus I (basket method). For formulation batches PM06, PM01A, PM01B, and PM01C, USP Apparatus II (paddle method) was employed. Dissolution testing was carried out in 900 mL of phosphate buffer (pH 6.8) maintained at 37 ± 0.5°C, with a rotation speed of 100 rpm. Six tablets from each formulation batch were individually introduced into round-bottom dissolution vessels at 5-minute intervals, ensuring sink conditions throughout the study. For formulations PM01–PM05, 10 mL aliquots were collected at 0 minute, 45 minutes, 2, 6, 10, and 12 hours. Sampling for PM01A–PM01C and the reference (innovator) product was performed at 0 minute, 45 minutes, 2, 6, 10, 12, 16, 18, 21, and 24 hours, while the control (PM06) was sampled at 5, 15, 30, 45, and 60 minutes. Withdrawn volumes were immediately replaced with equal volumes of fresh, pre-warmed dissolution medium to maintain constant volume and temperature. Each sample was filtered through a 0.22 µm membrane filter and analysed using a validated high-performance liquid chromatography (HPLC) method. The cumulative release of diclofenac sodium was calculated and plotted against time to generate dissolution profiles.

### 2.7. Determination of the difference (*f*_*1*_) and similarity (*f*_*2*_) factors

A model-independent approach was employed to evaluate the similarity between the dissolution profiles of the optimised formulation batches (PM01A–PM01C) and that of the innovator diclofenac sodium product, using the difference factor (*f₁*) and similarity factor (*f₂*) as statistical tools [14–16].

The difference factor (*f₁*) and similarity factor (*f₂*) were calculated using the following equations:


$$\text{Difference}\;\;\text{factor}\;(f_{\text{1}})\;=\;\frac{\Sigma \;(\text{Rt}-\text{Tt})}{\Sigma \;(\text{Rt})}x\text{1}\text{0}\text{0}$$ (10)

$$\text{Similarity}\;\;\text{factor}\;(f_{\text{2}})\;=\;\text{5}\text{0}\;x\;log\;[\;[\text{1}+\;\left(\frac{\text{1}}{n}x\Sigma \;(\text{Rt}-\text{Tt})\text{2}\;\right){]}^{-\text{0}.\text{5}\;}x\;\text{1}\text{0}\text{0}]$$ (11)

Where:

n = number of time points

Rt = cumulative percentage of diclofenac sodium released at time t from the innovator brand

Tt = cumulative percentage of diclofenac sodium released at time t from the optimized formulation batches

### 2.8. Kinetics and mechanism of diclofenac release from optimised matrix tablets (PM01A – PM01C)

#### 2.8.1. Zero-order drug release kinetics for diclofenac sodium release from PM01A-PM01C.

This was evaluated by plotting the mean cumulative percentage of diclofenac sodium released versus time. A linear relationship in this plot indicates zero-order release behaviour. The slope and correlation coefficient (R²) of the linear regression were used to assess the fit of the data to the model. The zero-order kinetic model is described by the equation [17,18]:


$$\;{\text{Q}}_{\text{t}}=\;{\text{K}}_{\text{0}}\;\text{t}$$ (12)

Where:

*Qₜ* = the amount of diclofenac sodium released at time *t*

*K₀* = the zero-order release rate constant

**t* *= the time

#### 2.8.2. First-Order drug release kinetics for diclofenac sodium release from PM01A-PM01C.

This was assessed by plotting the logarithm of the mean cumulative percentage of diclofenac sodium released versus time. The slope and correlation coefficient (R²) of the resulting linear plot were used to evaluate the fit of the data to the first-order model. The model is described by the following equation [19]:


$$\text{Log}\;\text{C}\;=\;\text{log}{\text{C}}_{\text{o}}\;\;–\;\text{kt}\;/\;\text{2}.\text{303}$$ (13)

Where:

*C* is the amount of diclofenac sodium released at time t

*C₀* is the initial concentration of diclofenac sodium

*k* is the first-order release rate constant

*t* is time

#### 2.8.3. Higuchi model of drug release kinetics for diclofenac sodium release from PM01A-PM01C.

The Higuchi model was used to describe drug release based on the diffusion mechanism. It was evaluated by plotting the mean cumulative percentage of drug released against the square root of time. The slope and correlation coefficient (R²) of the linear regression were used to assess the goodness of fit to the model. The Higuchi equation is expressed as [20]:


$$\text{Q}\;={\text{K}}_{\text{H}}{\text{t}}^{\text{1}/\text{2}}\;$$ (14)

Where:

*Q* – the amount of diclofenac sodium released at time *t*

*K*_*H*_ – the Higuchi release rate constant

*t* – time

#### 2.8.4. Hixson–Crowell model of drug release kinetics for diclofenac sodium release from PM01A-PM01C.

The Hixson–Crowell model was applied to assess diclofenac sodium release involving changes in surface area and diameter of the matrix tablets during dissolution. This was evaluated by plotting the cube root of the unreleased drug amount (or the cube root of the mean cumulative drug release) versus time. The slope and correlation coefficient (R²) of the linear plot were used to determine the model fit. The Hixson–Crowell equation is given as [17]:


$${\text{Q}}_{\text{0}}^{\text{1}/\text{3}}-{\text{Qt}}^{\text{1}/\text{3}}={\text{K}}_{\text{HC}}\text{t}\;$$ (15)

Where:

*Q₀* - the initial amount of diclofenac sodium in the matrix tablet

*Qₜ* – the amount of diclofenac sodium remaining at time *t*

*K*_*HC*_ – the Hixson–Crowell release rate constant

*t* – time

#### 2.8.5. Korsemeyer–Peppas model of drug release kinetics for diclofenac sodium release from PM01A-PM01C.

The Korsmeyer–Peppas model was applied to analyse the drug release mechanism from the polymeric matrix. This was done by plotting the logarithm of the mean cumulative percentage of diclofenac sodium released against the logarithm of time. The slope and correlation coefficient (R²) of the linear portion of the plot were used to evaluate the model fit. The model is described by the following equation [17,21]:


$${\text{M}}_{\text{t}}\;/\;\text{MT}\;=\;{\text{Kt}}^{\text{n}}$$ (16)

Where:

*Mₜ/Mₜ* – the fraction of drug released at time *t*

*K* – the kinetic constant incorporating structural and geometric characteristics of the dosage form

*t* – the release time

*n* – the diffusion exponent indicative of the release mechanism

### 2.9. Statistical analysis

Data obtained from the study were analysed using Microsoft Excel and GraphPad Prism version 10 (GraphPad Software, San Diego, CA, USA).

## 3.0. Results and discussion

### 3.1 FTIR compatibility test

Drug-excipient compatibility studies are important in formulation science. Drug-excipient interactions may affect the physicochemical properties, shelf stability, bioavailability, and, therefore, the efficacy and safety profile of the drug. Among other methods, the use of FTIR spectroscopy to identify and comparatively analyse the functional group compositions of drugs and excipients before and after formulation has become commonplace [22,23].

**Fig 2:**
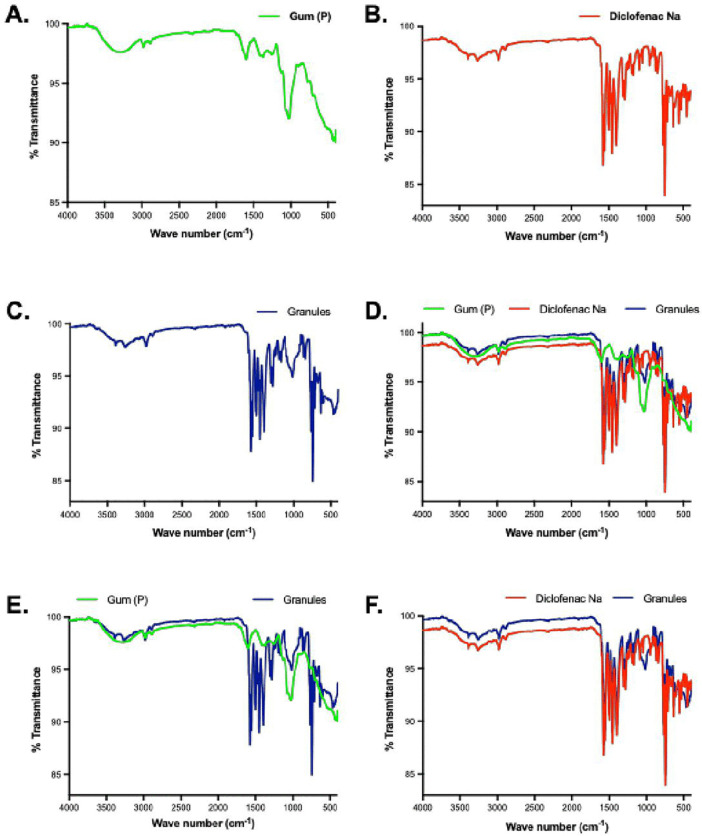
FT-IR spectrum of A. *Pentadesma butyracea* gum (P, green), B. diclofenac sodium (red), C. granules (blue) consisting of diclofenac sodium, talc, MCC and magnesium stereate, D gum, diclofenac sodium and granules overlaid on each other; spectrum shows distinct pattern for all three materials E. gum and granules overlaid on each other, showing significant differences, and F. diclofenac sodium and granules overlaid; spectrum of granules very similar to that of diclofenac sodium indicating compatibility.

The FT-IR spectrum of *Pentadesma butyracea* gum displayed functional group bands consistent with the secondary metabolites, including glycosides, identified through phytochemical screening. The bands consist of an O-H intermolecular bond stretch from 3590 cm^-1^ to 2990 cm^-1^ and its corresponding O-H bending at 1374 cm^-1^; C-H stretching of alkane at 2981 cm^-1^; C = C stretch at 1600 cm^-1^; and glycosidic C-O-C stretching and alcoholic C-O stretching at 1253 cm^-1^ and 1028 cm-1, respectively (Fig 2A; Table 4). Similar functional groups can be found in other gums, like guar, almond and grewia, which are of pharmaceutical interest [24–26].

**Table 4:** Characteristic FT-IR peaks for gum, diclofenac sodium and granules.

Material	Relevant peaks (cm^-1^)	Type of bond
*Pentadesma butyracea* gum (P)	1028	C-O stretch of primary alcohol
	1253	Glycosidic C-O stretching
	1374	O-H bending vibrations
	1600	C = C stretch
	2981	C-H stretching (alkane)
	3300 (2990–3590)	O-H stretching
Diclofenac Sodium	746	C-Cl bending vibrations
	952	C = C bending of alkene
	1305	C-N stretching
	1398	O-H bending
	1453	Aromatic C-H bending
	1498,1557	Aromatic C = C stretching
	1574	Asymmetrical COO^-^ stretching of carboxylic acid
	2981	C-H stretching
	3250	O-H stretching of carboxylic acid
	3388	N-H stretching
Granules	671	O-H bending of talc
	745	C-Cl bending vibrations
	1013	Si-O bending from excipients
	1160	asymmetric C-O-C stretching vibration (MCC)
	1304	C-N stretching
	1385	O-H bending
	1452	Aromatic C-H bending
	1498,1556	Aromatic C = C stretching
	1572	COO^-^ stretching
	2981	C-H stretching
	3255	O-H stretching of carboxylic acid
	3389	N-H stretching

For diclofenac sodium, IR peaks were detected for N-H and C-H stretching vibrations at 3388 cm^-1^ and 2981 cm^-1^ for respectively. C-N stretching was at 1305 cm^-1^. There was a broad O-H stretching vibration from carboxylic acid, which peaks at 3250 cm^-1^, while the asymmetric stretching of carboxylate ion was at 1574 cm^-1^. The corresponding O-H bending showed at 1398 cm^-1^. Double bond vibrations like aromatic C = C stretching were detected at 1557 cm^-1^ and 1498 cm^-1^, and the C = C bending vibrations were at 952 cm^-1^. The *sp*^*2*^ hybridised C-H bending occurred at 1470 cm^-1^ and 1453 cm^-1^. C-Cl bending vibrations were at 746 cm^-1^ (Fig 2B; Table 4). These bands have been extensively reported [27].

The granules were formulated using *Pentadesma butyracea* gum, MCC, talc and magnesium stearate as the excipients and diclofenac sodium as the API. Characteristic peaks for talc are found at 1013 cm^-1^ (Si-O bending) and 671 cm^-1^ (O-H bending) [28]. C-H stretching vibrations occurred at 2981 cm^-1^–2850 cm^-1^. O-H stretching was observed at 3250 cm^-1^ while O-H bending was at 1385 cm^-1^. Infrared absorption due to asymmetric C-O-C stretching of MCC occurred at 1160 cm^-1^ (Fig 2C; Table 4) [29]. Besides those peaks in the IR spectrum of the granules accounted for by the excipients, the other peaks are accounted for the peaks already discussed for diclofenac (Fig 2F; Table 4).

Drug and excipients are said to be incompatible when the FTIR data of formulation, in comparison to that of the drug, indicate either the appearance of new peaks, significant shifts in the peak positions, or a loss or decrease in peak intensity. Comparing the IR spectra of the *Pentadesma butyracea* gum, diclofenac sodium, and granules highlights some similarities and differences in functional groups (Fig 2D). For instance, the *Pendesma butyracea* gum shows a spectrum profile distinct from that of the granules (Fig 2D and 2E), with the overlapping peaks at 1028 cm^-1^ and 1013 cm^-1^ due to different functional groups in the gum and the granules, respectively. The IR spectrum of diclofenac and the granules (formulation) showed very similar peaks. Characteristic peaks for the excipients also showed in the granules. A shift in the spectrum baseline of the granules may account for the slight change in peak intensities (Fig 2F). In conclusion, as there was no significant change in functional groups of the excipients and the API in granules, the excipients may be said to be compatible with diclofenac sodium and safe for use in formulation.

### 3.2. The flow properties of the diclofenac sodium granules before compression

To mitigate issues such as flow obstruction and irregular flow during granule compression, the flow properties of each batch were assessed. According to [8], Carr’s Compressibility index values ranging from 5–11%, 12–16%, 18–21%, 23–25%, 33–38%, and > 40% are indicative of excellent, good, fair, poor, very poor, and extremely poor flowability, respectively. Similarly, a Hausner’s ratio below 1.2 is associated with good flow characteristics, whereas values ≥ 1.5 suggest poor flowability. The angle of repose further categorises flowability, with values of 25–30°, 31–35°, 36–40°, and > 46° corresponding to excellent, good, passable, and very poor flow, respectively. The results, as presented in Table 5, reveal that the granules of batches PM01, PM02, PM03, PM05 and PM06 exhibited excellent flow properties. However, batches PM04, PM01A, PM01B, and PM01C demonstrated good flowability. These findings indicate that the granules possessed adequate flow properties suitable for compression into tablets.

**Table 5:** Precompressional evaluation of granules.

Formulation code	Bulk density (mg/mL)	Tapped density (mg/mL)	Hausner ratio	Compressibility index (%)	Angle of repose (º)
PM01	0.496 ± 0.008	0.550 ± 0.010	1.11 ± 0.002	10.96 ± 0.212	30.65 ± 0.263
PM02	0.536 ± 0.007	0.582 ± 0.009	1.09 ± 0.002	8.70 ± 0.178	30.11 ± 0.510
PM03	0.424 ± 0.004	0.457 ± 0.002	1.08 ± 0.016	7.96 ± 1.607	30.48 ± 1.042
PM04	0.414 ± 0.004	0.462 ± 0.006	1.12 ± 0.002	11.50 ± 0.187	31.41 ± 0.275
PM05	0.460 ± 0.008	0.507 ± 0.009	1.10 ± 0.002	10.13 ± 0.181	30.11 ± 0.510
PM06	0.375 ± 0.004	0.427 ± 0.028	1.14 ± 0.085	10.96 ± 0.212	29.75 ± 0.000
PM01A	0.483 ± 0.025	0.558 ± 0.02	1.156 ± 0.018	15.57 ± 1.811	34.14 ± 0.636
PM01B	0.540 ± 0.012	0.620 ± 0.005	1.147 ± 0.016	14.72 ± 1.608	32.01 ± 0
PM01C	0.466 ± 0.008	0.527 ± 0.002	1.132 ± 0.035	13.18 ± 1.352	32.42 ± 0.583

### 3.3. Uniformity in the thickness and diameter of formulations PM01 – PM06; PM01A – PM01C

The weight of a tablet is primarily determined by the density of the powder blend, alongside the diameter and thickness of the final dosage form. Theoretically, tablet diameter remains constant, as it is governed by the fixed dimensions of the punches and dies employed during compression. As shown in Table 6, the mean tablet diameter across all formulation batches ranged between almost 12 mm to 12.07 mm. These slight deviations are likely due to minor surface irregularities or wear of the punches and dies during the compression process [9]. Tablet thickness uniformity serves as an essential quality control criterion, ensuring consistent physical appearance and facilitating efficient packaging. Moreover, uniform thickness contributes to the accurate operation of automated tablet counting and filling equipment. Regular monitoring of tablet thickness during manufacturing may allow for early detection of weight or content non-uniformity. Data presented in Table 6 indicate that tablet thickness values ranged from 3.03 mm to 3.14 mm across all batches. All compressed tablets met the British Pharmacopoeia [6] specification for dimensional uniformity, with no single tablet exhibiting more than ± 5% deviation in either thickness or diameter. This consistency is attributable to the acceptable flow properties of the granules, which likely enabled uniform die filling during the compression process.

**Table 6:** Uniformity of weight, tablet dimension of formulations and diclofenac sodium content in PM01 – PM06; PM01A – PM01C.

Formulation code	Mean weight of tablets (n = 20)/ g	No. of tablets deviated by ≥ ± 5%	No. of tablets deviated by ± 10%	Mean diameter/mm (n = 10)	Mean thickness/ mm (n = 10)	% Content of diclofenac sodium (n = 3)
PM01	0.416 ± 0.005	0	0	12.04 ± 0.041	3.07 ± 0.076	100.45 ± 0.624
PM02	0.408 ± 0.005	0	0	12.06 ± 0.036	3.05 ± 0.023	101.21 ± 2.779
PM03	0.417 ± 0.005	0	0	12.05 ± 0.037	3.03 ± 0.023	100.52 ± 1.659
PM04	0.413 ± 0.006	0	0	12.04 ± 0.036	3.03 ± 0.037	100.82 ± 1.757
PM05	0.413 ± 0.004	0	0	12.07 ± 0.034	3.04 ± 0.039	101.87 ± 2.542
PM06	0.413 ± 0.006	0	0	12.05 ± 0.033	3.06 ± 0.038	101.21 ± 2.485
PM01A	0.406 ± 0.004	0	0	11.98 ± 0.001	3.21 ± 0.036	99.67 ± 0.606
PM01B	0.404 ± 0.003	0	0	12.04 ± 0.136	3.14 ± 0.028	100.06 ± 0.288
PM01C	0.405 ± 0.003	0	0	12.06 ± 0.153	3.14 ± 0.05	99.11 ± 0.587

### 3.4. Weight variation of formulations PM01 – PM06; PM01A – PM01C

The weight variation test serves as an indirect measure of the uniformity of drug content within a batch of tablets, as tablet weight correlates with the quantity of active pharmaceutical ingredient (API) it may contain. Achieving consistent tablet weight is therefore essential to ensure accurate dosing, which in turn contributes to attaining the desired therapeutic drug concentration and clinical efficacy. In the present study, the average weight of all matrix tablet batches exceeded 250 mg. According to the British Pharmacopoeia [6], for tablets with an average weight greater than 250 mg, not more than two out of twenty randomly selected tablets should deviate by more than ± 5% from the mean weight, and none should deviate by more than ± 10%. As presented in Table 6, all formulation batches complied with these specifications, indicating satisfactory uniformity of weight. The observed compliance may be attributed to the favourable flow characteristics of the granules, uniform die filling, and consistent movement of the lower punch during compression. Consequently, the tablets are expected to exhibit minimal variation in drug content, supporting consistent therapeutic performance across individual units.

### 3.5 Diclofenac sodium content in formulations PM01 – PM06; PM01A – PM01C

The assay of pharmaceutical products is a critical quality control parameter, as it confirms the presence and quantifies the content of the active pharmaceutical ingredient (API) in dosage forms. It also plays a vital role in identifying and eliminating substandard and counterfeit products from the market. Based on the results presented (Table 6), all formulated batches complied with pharmacopoeial specifications, with diclofenac sodium content ranging from 100.21% to 100.82%, which falls within the United States Pharmacopoeia [30], acceptable range of 90–110%. These findings indicate not only the presence of diclofenac sodium in the matrix tablet formulations but also confirm that each batch contains the appropriate quantity necessary to ensure consistent therapeutic efficacy.

### 3.6. Mechanical integrity of formulations PM01 – PM06; PM01A – PM01C

#### 3.6.1. Hardness of formulations PM01 – PM06; PM01A – PM01C.

Tablet hardness refers to the ability of a tablet to resists breaking or crushing under mechanical stress encountered during manufacturing and use, thereby offering insight into the tablet’s structural robustness. As a critical quality control parameter, hardness is routinely evaluated during the compression process, as the data obtained informs necessary adjustments to the compression force applied by the tableting machine. Tablets with insufficient hardness may lack the mechanical integrity to withstand subsequent manufacturing steps such as coating and packaging, whereas overly hard tablets may fail to disintegrate within the required time frame, potentially compromising dissolution and bioavailability. A minimum crushing strength of 4 kgf is generally considered acceptable for ensuring mechanical stability without compromising disintegration performance [6]. As presented in Table 7, all the formulation batches exhibited hardness values within the acceptable range (4–10 kgf), thereby passing the hardness test. This outcome may be attributed to the application of an appropriate and consistent compression force during tablet manufacture [31].

**Table 7:** Mechanical strength of formulation batches PM01 – PM06; PM01A-PM01C.

Formulation code	Hardness/ kgf (n = 5)	Friability/% (n = 3)	Tensile strength/ MPa
PM01	8.13 ± 0.849	0.66 ± 0.031	1.37 ± 0.133
PM02	7.74 ± 0.897	0.61 ± 0.040	1.31 ± 0.155
PM03	7.52 ± 0.315	0.17 ± 0.020	1.29 ± 0.054
PM04	8.08 ± 0.777	0.34 ± 0.040	1.39 ± 0.131
PM05	8.73 ± 0.806	0.27 ± 0.020	1.49 ± 0.141
PM06	8.60 ± 0.834	0.46 ± 0.020	1.46 ± 0.143
PM01A	8.54 ± 0.379	0.79 ± 0.010	1.41 ± 0.060
PM01B	8.44 ± 0.572	0.5 ± 0.010	1.42 ± 0.109
PM01C	8.83 ± 0.356	0.25 ± 0.012	1.48 ± 0.047

#### 3.6.2. Friability of formulations PM01 – PM06; PM01A – PM01C.

Tablet friability evaluates the mechanical integrity of tablets by determining their resistance to abrasion and breakage under stress, thereby indicating their ability to endure subsequent processing, handling, packaging, and transportation. According to [6], a weight loss of not more than 1% is considered acceptable for conventional tablets; values exceeding this threshold indicate inadequate mechanical robustness. As shown in Table 7, all formulation batches passed the friability test. This implies that PM01-PM06 and PM01A-PM01C tablets could resist physical defects such as chipping, crumbling, capping, or breakage.

#### 3.6.3. Tensile strength of formulations PM01 – PM06; PM01A – PM01C.

Tensile strength is a critical mechanical property of tablet formulations. In this study, the tensile strength of the formulated batches ranged from 1.29 ± 0.054 to 1.49 ± 0.141 MPa. For small-scale tablet production, a tensile strength of ≥ 1 MPa is generally considered acceptable to ensure adequate mechanical robustness [32,33]. Based on this criterion, all formulations met the required specification (Table 7). This means that all formulation batches have sufficient balance between structural integrity to withstand handling and adequate friability to allow disintegration and dissolution.

### 3.7. *In-vitro* diclofenac release from formulation batches PM01-PM06; PM01A–PM01C

#### 3.7.1. HPLC method validation for the quantitative analysis of diclofenac sodium in dissolution testing.

**Fig 3:**
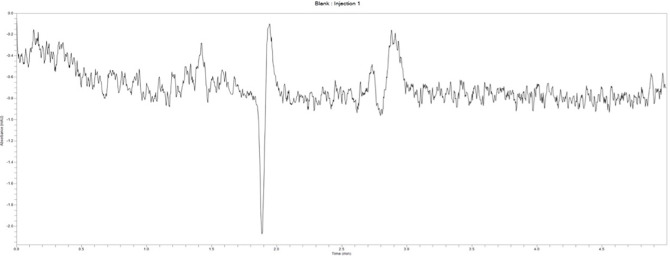
chromatogram for blank.

**Fig 4:**
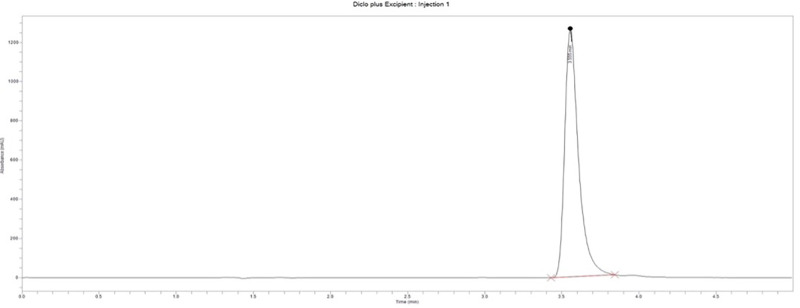
Chromatogram for diclofenac sodium tablets without matrixing agent.

**Fig 5:**
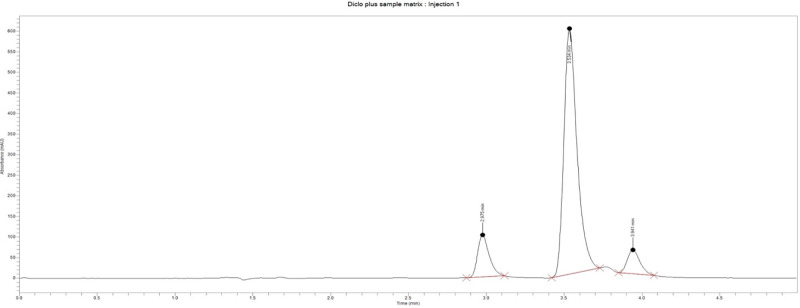
chromatogram for diclofenac sodium matrix tablet.

***Specificity.*** This test was performed to verify that the developed HPLC method showed no interference from excipients in the formulation. Diclofenac sodium exhibited an average retention time of 3.50 ± 0.016 minutes at 254 nm. The blank displayed no peaks within the run time (Fig 3), indicating a high level of specificity. The conventional release diclofenac tablet (PM06) produced a peak with an area of 7,846,913.9 mAU at a retention time of 3.55 minutes (Fig 4). The matrix formulation also displayed three peaks with areas of 524,661.8 mAU, 3,501,481.2 mAU, and 325,747.4 mAU at retention times of 2.975 minutes, 3.534 minutes, and 3.941 minutes, respectively (Fig 5). The diclofenac sodium peak was observed at a retention time of 3.534 minutes, while the other peaks could be characteristic of compounds from *Pentadesma butyracea* gum.

**Fig 6:**
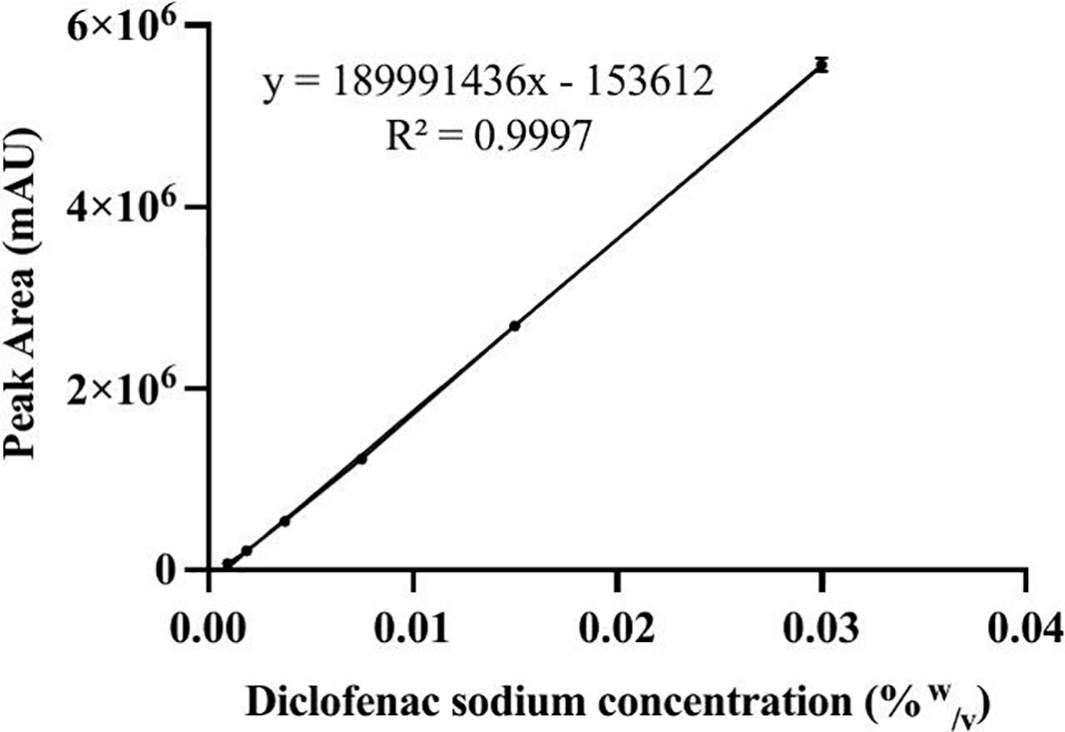
Calibration plot for diclofenac sodium in phosphate buffer of pH 6.8.

***Linearity, limit of detection (LOD) and limit of quantification (LOQ).*** The calibration curve plotted from concentration 0.0009375%^w^/_v_ to 0.03%^w^/_v_ was linear with a correlation coefficient (R^2^) of 0.9997 (Fig 6) for the line equation, y = 189991436x – 153612. The obtained R^2^ value indicates a strong positive correlation between the diclofenac sodium concentration and the peak area; thus, as the concentration decreases, the peak area also decreases. The LOQ and LOD were determined to be 0.001790723%^w^/_v_ and 0.00059094%^w^/_v,_ respectively.

***Precision and accuracy.*** This method was precise, with a relative standard deviation (RSD) value of 0.5800% for freshly prepared samples and 0.4113% for intraday precision (Table 8). This method also demonstrated RSD values of 0.2289%, 0.5756%, and 0.3305% for inter-day precision on days 1, 2, and 3 (Table 9). Since the obtained RSD values were < 2, it indicates that the developed analytical method is reproducible, consistent, and reliable [34].

**Table 8:** Average percentage recoveries and Relative Standard Deviation (RSD) values for precision of diclofenac sodium at 254 nm.

Sample condition	Prepared concentration (µg/ mL)	Amount of recovered diclofenac sodium (µg/ mL) (n = 6)	% Mean recovery (n = 6)	RSD/ %
Freshly prepared	150	148.124 ± 0.859	98.75 ± 0.573	0.5800
Intra day	150	147.454 ± 0.606	98.30 ± 0.404	0.4113

**Table 9:** Mean percentage recoveries and Relative Standard Deviation values of intermediate precision of diclofenac sodium at 254 nm.

Day	Prepared concentration (µg/ mL)	Amount of recovered diclofenac sodium (µg/ mL) (n = 3)	% Mean recovery (n = 3)	RSD/ %
1	150	152.281 ± 0.349	101.52 ± 0.232	0.2289
2	150	152.491 ± 0.878	101.66 ± 0.585	0.5756
3	150	152.665 ± 0.505	101.78 ± 0.336	0.3305

The accuracy of the developed HPLC method was evaluated by determining the closeness of measured values to their corresponding theoretical concentrations, expressed as the percentage mean recovery of diclofenac sodium. The recovery values ranged from 96.71 ± 0.39% to 100.29 ± 1.20% (Table 10), which falls within the acceptable range of 90–110% as recommended by [35]. These findings confirm that the developed method is capable of delivering accurate quantification of diclofenac sodium and is therefore suitable for application in quality control analyses.

**Table 10:** Mean % diclofenac sodium recovery at wavelength 254 nm.

Sample	Prepared concentrations (%^w^/_v_)	Concentration (µg/ mL)	Amount of recovered diclofenac sodium (µg/ mL)	Average diclofenac sodium recovered (%) (n = 3)
Diclofenac sodium	0.03	300	300.875 ± 3.611	100.29 ± 1.204
	0.015	150	149.549 ± 0.933	99.70 ± 0.622
	0.0075	75	72.532 ± 0.294	96.71 ± 0.3915

***Robustness.*** Robustness evaluates the reliability of an analytical method under small, deliberate variations in procedural parameters. The percentage mean recoveries obtained ranged from 99.85 ± 0.046% to 101.04 ± 0.066%, with relative standard deviations (RSD) below 2%. These results indicate no statistically significant differences (p > 0.05) in the chromatographic responses, thereby confirming the robustness of the developed HPLC method (Table 11).

**Table 11:** Mean percentage recoveries and percentage Relative Standard Deviation values for column and mobile phase variation at 254 nm.

Prepared concentration (µg/ mL)	Amount of recovered diclofenac sodium (µg/ mL) (n = 3)	% Mean recovery (n = 3)	RSD/ %
300	299.560 ± 0.139	99.85 ± 0.046	0.0464
150	150.430 ± 0.274	100.29 ± 0.183	0.1825
75	75.778 ± 0.050	101.038 ± 0.066	0.0656

#### 3.7.2. Assessment of *Pentadesma butyracea* gum as a drug release modifier.

**Fig 7:**
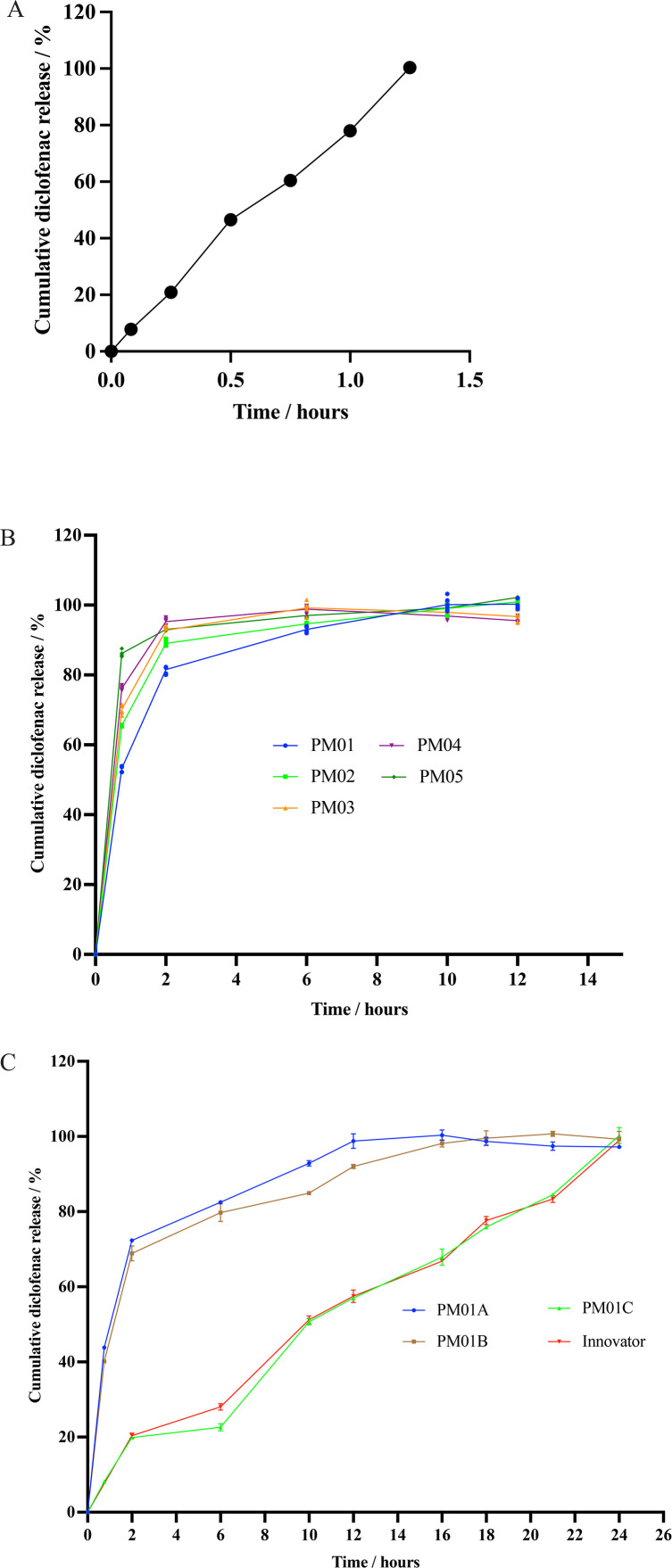
*In vitro* diclofenac sodium release from non-matrix formulation (A), matrix formulations (B), optimized matrix formulations and innovator brand of diclofenac sodium retard tablets (C).

The developed matrix tablets were intended to facilitate the extended release of diclofenac sodium, ideally over 24 hours. The drug release-retarding potential of *Pentadesma butyracea* gum was evaluated by comparing diclofenac sodium release from formulations containing the gum as the sole matrix former (PM01–PM05) with a non-matrix control formulation (PM06). PM06 failed the dissolution test for conventional-release tablets, releasing only 60.46 ± 0.201% of the drug (Fig 7A), which is below the pharmacopeial threshold of 70% [6]. This failure may be attributed to the absence of a disintegrant in the formulation.

An increase in *P. butyracea* gum concentration beyond 15%^w^/_w_ led to a gradual, time-dependent increase in diclofenac sodium release (Fig 7B). At 45 minutes, more than 50% of diclofenac sodium had been released in all batches, with PM01 releasing the lowest amount, but failing to meet the British Pharmacopoeia requirements for modified-release formulations.

To achieve a compliant extended-release profile, PM01 was optimised by incorporating varying concentrations of xanthan gum. Among the optimised formulations, PM01C, containing 15%^w^/_w_ *P. butyracea* gum and 20%^w^/_w_ xanthan gum, demonstrated 19.90 ± 0.508% drug release at 2 hours and 100.26 ± 2.118% at 24 hours (Fig 7C). This conforms to [6] specification requiring 10–30% release within 2 hours and ≥ 80% after 24 hours. These results indicate that the combination of 15%^w^/_w_ *P. butyracea* gum and ≥ 20%^w^/_w_ xanthan gum is effective in modulating diclofenac sodium release over 24 hours.

#### 3.7.3. Comparison of dissolution profiles: similarity (f_2_) and difference (f_1_) factors.

Comparative *in vitro* dissolution studies between the innovator product and the optimised formulations (PM01A, PM01B, and PM01C) were conducted using the difference factor (*f₁*) and similarity factor (*f₂*) as described by [36–38]. Formulation PM01C demonstrated *f₁* and *f₂* values of 2.93 and 79.34, respectively, which fall within the acceptable ranges (*f₁*: 0–15; *f₂:* 50–100), indicating a high level of similarity to the reference formulation. In contrast, PM01A and PM01B exhibited *f₁* and *f₂* values outside these limits, signifying notable differences in their dissolution profiles compared to the innovator product (Table 12) [39].

**Table 12:** Difference and similarity factors between the optimised matrix tablets and the innovator brand.

Formulation code	PM01A	PM01B	PM01C
Difference factor (f_1_)	53.68	49.68	2.93
Similarity factor (f_2_)	21.66	23.56	79.34
Comment	Different	Different	Similar

#### 3.7.4. Investigation of the release kinetics and mechanism of diclofenac sodium from optimised matrix formulations through mathematical modelling.

The *in vitro* dissolution profile was employed to mathematically evaluate the kinetics and mechanism of diclofenac sodium release from the optimised matrix tablets. The coefficient of determination (R²) provided statistical insight into the best-fitting drug release model (Table 13). Formulation PM01C followed zero-order kinetics, exhibiting an R² value of 0.9819, indicative of a constant drug release rate independent of the remaining drug concentration within the matrix. This kinetic behaviour is ideal for matrix-based formulations, as it supports the maintenance of consistent serum drug levels throughout the delivery period [18,40]. In contrast, formulations PM01A and PM01B followed the Korsmeyer-Peppas release model, with R² values of 0.8885 and 0.9198, respectively. This suggests that drug release from these formulations occurs primarily by diffusion, analogous to release from polymeric films [21,41].

**Table 13:** Kinetics and mechanism of diclofenac sodium release from developed optimised matrix tablets.

Formulation code	Zero order	First order	Higuchi	Hixson-Crowell	Korsmeyer-Peppas	n
	R^2^					
PM01A	0.6406	0.6647	0.2160	0.5688	0.8885	0.2160
PM01B	0.7619	0.8743	0.2421	0.7870	0.9198	0.2421
PM01C	0.9819	0.9211	0.6941	0.7425	0.9572	0.6941

The mechanism of drug release was further elucidated using the release exponent (n), derived from the slope of the log cumulative percentage drug release versus log time plot. According to established classification, an n-value ≤ 0.45 indicates Fickian diffusion, 0.45 < n < 0.89 suggests non-Fickian (anomalous) transport, and n > 0.89 corresponds to super case II transport. PM01A and PM01B demonstrated Fickian diffusion, indicating diffusion-controlled drug release, while PM01C exhibited non-Fickian diffusion, suggesting a combination of diffusion and polymer relaxation as the dominant release mechanism [42,43]. The observed differences in drug release kinetics and mechanisms among the formulations may be attributed to variations in polymer concentration, influencing the swelling and degradation behaviour of the matrix and thereby altering its physicochemical characteristics [44].

## 4.0. Conclusion

The use of purified *Pentadesma butyracea* gum alone was insufficient to sustain the release of diclofenac sodium from matrix tablet formulations, particularly at higher gum concentrations. However, the incorporation of xanthan gum, at the lowest concentration of *P. butyracea* gum evaluated, markedly enhanced the drug release retardation capacity of the matrix. Among the optimised formulations, PM01C demonstrated superior performance as an extended-release matrix system for diclofenac sodium. This formulation exhibited a drug release profile comparable to that of the innovator brand and conformed to zero-order release kinetics with a non-Fickian diffusion mechanism. These results suggest that a combination of xanthan gum at concentrations ≥ 20%^w^/_w_ with 15%^w^/_w_ *P. butyracea* gum represents a promising hydrophilic matrix carrier system for achieving extended release of diclofenac sodium.

## Supporting information

S1 File Appendices. (DOCX)

## References

[1] Maderuelo C, Zarzuelo A, Lanao JM. Critical factors in the release of drugs from sustained release hydrophilic matrices. J Control Release. 2011;154(1):2–19. doi: 10.1016/j.jconrel.2011.04.002 21497624

[2] Alqahtani MS, Kazi M, Alsenaidy MA, Ahmad MZ. Advances in Oral Drug Delivery. Front Pharmacol. 2021;12:618411. doi: 10.3389/fphar.2021.618411 33679401 PMC7933596

[3] Prasad V, De Jesús K, Mailankody S. The high price of anticancer drugs: origins, implications, barriers, solutions. Nat Rev Clin Oncol. 2017;14(6):381–90. doi: 10.1038/nrclinonc.2017.31 28290490

[4] Nagendrakumar D, Keshavshetti GG, Shardor AG. An overview: Matrix tablets as sustained release. Recent Research in Science and Technology. 2014;5(4).

[5] Kamaly N, Yameen B, Wu J, Farokhzad OC. Degradable Controlled-Release Polymers and Polymeric Nanoparticles: Mechanisms of Controlling Drug Release. Chem Rev. 2016;116(4):2602–63. doi: 10.1021/acs.chemrev.5b00346 26854975 PMC5509216

[6] British Pharmacopoeia. British Pharmacopoeia. Her Majesty’s Stationary Office. 2021.

[7] Indian Pharmacopoeia Commission. Indian Pharmacopoeia. Vols. I–III. Government of India, Ministry of Health and Family Welfare.; 2018.

[8] Aulton ME, Taylor KMG. Aulton’s Pharmaceutics E-Book: The Design and Manufacture of Medicines. Elsevier Health Sciences. 2017.

[9] Fosu MA, Ofori-Kwakye K, Kuntworbe N, Bonsu MA. Investigation of blends of cashew and xanthan gums as a potential carrier for colonic delivery of ibuprofen. Int J Pharmtech Res. 2016;9(7):369–80.

[10] Snyder LR, Kirkland JJ, Dolan JW. Introduction to modern liquid chromatography. John Wiley & Sons. 2011.

[11] Pērkons I. The optimization of analytical parameters for screening and quantification of pharmaceutical residues in the environment [Internet] [PhD Thesis]. University of Latvia; 2021 [cited 2025 Nov 13]. Available from: https://dspace.lu.lv/handle/7/54189

[12] Kazakevich YV, Lobrutto R. HPLC for pharmaceutical scientists. John Wiley & Sons; 2006.

[13] ICH Guideline. Validation of analytical procedures: text and methodology. Q2 (R1). 2005;1(20):05.

[14] Kollipara S, Boddu R, Ahmed T, Chachad S. Simplified Model-Dependent and Model-Independent Approaches for Dissolution Profile Comparison for Oral Products: Regulatory Perspective for Generic Product Development. AAPS PharmSciTech. 2022;23(1):53. doi: 10.1208/s12249-021-02203-7 35028797

[15] Moore JW, Flanner HH. Mathematical comparison of dissolution profiles. Pharmaceutical technology. 1996;20(6):64–74.

[16] Shah VP, Lesko LJ, Fan J, Fleischer N, Handerson J, Malinowski H, et al. fDA Guidance for Industry 1 Dissolution Testing of Immediate Release Solid Oral Dosage Forms. Dissolution Technol. 1997;4(4):15–22. doi: 10.14227/dt040497p15

[17] Singhvi G, Singh M. In-vitro drug release characterization models. Int J Pharm Stud Res. 2011;2(1):77–84.

[18] Hadjiioannou TP, Christian GD, Koupparis MA, Macheras PE. Quantitative calculations in pharmaceutical practice and research. New York: VCH. 1993.

[19] Dash S, Murthy PN, Nath L, Chowdhury P. Kinetic modeling on drug release from controlled drug delivery systems. Acta Pol Pharm. 2010;67(3):217–23. 20524422

[20] Higuchi T. Mechanism of sustained-action medication. theoretical analysis of rate of release of solid drugs dispersed in solid matrices. J Pharm Sci. 1963;52:1145–9. doi: 10.1002/jps.2600521210 14088963

[21] Korsmeyer RW, Gurny R, Doelker E, Buri P, Peppas NA. Mechanisms of solute release from porous hydrophilic polymers. Int J Pharm. 1983;15(1):25–35. 10.1002/jps.2600721021 6644570

[22] Chadha R, Bhandari S. Drug-excipient compatibility screening--role of thermoanalytical and spectroscopic techniques. J Pharm Biomed Anal. 2014;87:82–97. doi: 10.1016/j.jpba.2013.06.016 23845418

[23] Bunaciu AA, Hoang VD, Aboul-Enein HY. Fourier transform infrared spectroscopy used in drug excipients compatibility studies. Appl Spectrosc Rev. 2025;60(5):385–403.

[24] Mudgil D, Barak S, Khatkar BS. X-ray diffraction, IR spectroscopy and thermal characterization of partially hydrolyzed guar gum. Int J Biol Macromol. 2012;50(4):1035–9. doi: 10.1016/j.ijbiomac.2012.02.031 22409871

[25] Nep EI, Conway BR. Preformulation studies on grewia gum as a formulation excipient. J Therm Anal Calorim. 2012;108(1):197–205.

[26] Bashir M, Haripriya S. Assessment of physical and structural characteristics of almond gum. Int J Biol Macromol. 2016;93(Pt A):476–82. doi: 10.1016/j.ijbiomac.2016.09.009 27608543

[27] Shivakumar HN, Desai BG, Deshmukh G. Design and optimization of diclofenac sodium controlled release solid dispersions by response surface methodology. Indian J Pharm Sci. 2008;70(1):22–30. doi: 10.4103/0250-474X.40327 20390076 PMC2852056

[28] Li X, Zhang Y, He Y. Rapid detection of talcum powder in tea using FT-IR spectroscopy coupled with chemometrics. Sci Rep. 2016;6:30313. doi: 10.1038/srep30313 27468701 PMC4965860

[29] Yong WS, Yeu YL, Chung PP, Soon KH. Extraction and characterization of microcrystalline cellulose (MCC) from durian rind for biocomposite application. J Polym Environ. 2024;32(12):6544–75.

[30] The United States Pharmacopeia. 34th ed. Rockville, Maryland: US Pharmacopeia Convention; 2011.

[31] Sacher S, Kottlan A, Diop J-B, Heimsten R. Prediction of in-vitro dissolution and tablet hardness from optical porosity measurements. Int J Pharm. 2024;660:124336. doi: 10.1016/j.ijpharm.2024.124336 38871136

[32] Jantarat C, Kaewpradit S, Chingunpitak J, Srivaro S. Characteristics of microcrystalline cellulose derived from oil palm trunk slabs and its potential for use as tablet diluent. Heliyon. 2025;11(4):e42902. doi: 10.1016/j.heliyon.2025.e42902 41477499 PMC11909442

[33] McCormick D. Evolutions in direct compression. Pharmaceutical technology. 2005;29(4):52–62.

[34] Kishore D, Prasad KRS, Darapureddy C, Phani RSCH. Development and validation of a new HPLC bioanalytical internal standard method for the analysis of remdesivirin human plasma. RJC. 2022;14(04):2639–44. doi: 10.31788/rjc.2021.1446373

[35] Alquadeib BT. Development and validation of a new HPLC analytical method for the determination of diclofenac in tablets. Saudi Pharm J. 2019;27(1):66–70. doi: 10.1016/j.jsps.2018.07.020 30662308 PMC6323142

[36] Gohel MC, Sarvaiya KG, Mehta NR, Soni CD, Vyas VU, Dave RK. Assessment of similarity factor using different weighting approaches. Dissolut Technol. 2005;12(4):22.

[37] Kulkarni VS, Butte KD, Rathod SS. Natural polymers–A comprehensive review. Int J Res Pharm Biomed Sci. 2012;3(4):1597–613.

[38] Archer M-A, Kumadoh D, Gaizer SN-B, Mensah A, Jato J, Kyene MO, et al. Development and In Vitro Evaluation of Oral Capsules from Antiaris: A Convenient Substitute for Peripheral Neuropathy. Adv Pharmacol Pharm Sci. 2022;2022:5340953. doi: 10.1155/2022/5340953 35528114 PMC9068321

[39] Akhtar H, Razvi N, Khan S, Khan K, Ullah S, Ansari RA. Application of Quality Control Parameters and Model Dependent and Independent Approaches on Different Brands of Itopride HCL 250mg Available in Karachi, Pakistan. Annals of Jinnah Sindh Medical University. 2025;11(1):9–14.

[40] Askarizadeh M, Esfandiari N, Honarvar B, Sajadian SA, Azdarpour A. Kinetic Modeling to Explain the Release of Medicine from Drug Delivery Systems. ChemBioEng Reviews. 2023;10(6):1006–49. doi: 10.1002/cben.202300027

[41] Talevi A, Ruiz ME. Korsmeyer-peppas, peppas-sahlin, and brazel-peppas: models of drug release. In: The ADME encyclopedia: A comprehensive guide on biopharmacy and pharmacokinetics. Springer; 2022. p. 613–21.

[42] Rahamathulla M, Alshahrani SM, Al Saqr A, Alshetaili A, Shakeel F. Effervescent floating matrix tablets of a novel anti-cancer drug neratinib for breast cancer treatment. Journal of Drug Delivery Science and Technology. 2021;66:102788. doi: 10.1016/j.jddst.2021.102788

[43] Talevi A, Ruiz ME. Korsmeyer-peppas, peppas-sahlin, and brazel-peppas: models of drug release. In: The ADME encyclopedia: A comprehensive guide on biopharmacy and pharmacokinetics. Springer; 2022. p. 613–21.

[44] Boakye-Gyasi ME, Owusu FWA, Entsie P, Agbenorhevi JK, Banful BKB, Bayor MT. Pectin from Okra (Abelmoschus esculentus L.) Has Potential as a Drug Release Modifier in Matrix Tablets. ScientificWorldJournal. 2021;2021:6672277. doi: 10.1155/2021/6672277 33531880 PMC7834820

