# Supplementary material for: Investigation of the drug release modifying property of Pentadesma butyracea gum in diclofenac sodium matrix tablets
Source: PLoS One. 2026 Jan 29;21(1):e0341668. doi: 10.1371/journal.pone.0341668 (PMC12854478; doi:10.1371/journal.pone.0341668)
Supplement: S1 File — (DOCX) [file pone.0341668.s001.docx]

**S1. APPENDICES**

**S1.1 Appendix. Physicomechanical properties of diclofenac sodium granules**

S1 Table. Flow properties of the granules

| Formulation code | Weight of granules | Bulk volume | Bulk density | Tapped volume | Tapped density | Hausner ratio | Carr’s index | Height | Radius of cone | Angle of repose |
| --- | --- | --- | --- | --- | --- | --- | --- | --- | --- | --- |
| PM01 | 10.187 | 10.187 | 27 | 0.3773 | 25 | 1.08 | 8 | 2 | 3.5 | 29.7449 |
|  | 10.05 | 10.05 | 27 | 0.3722 | 22.5 | 1.2 | 20 | 2 | 3.5 | 29.7449 |
|  |  |  |  |  |  |  |  |  |  |  |
| PM02 | 10.022 | 10.022 | 20 | 0.5011 | 18 | 1.1111 | 11.1111 | 2 | 3.4 | 30.4655 |
|  | 10.05 | 10.05 | 20.5 | 0.4902 | 18.5 | 1.1081 | 10.8108 | 2 | 3.5 | 29.7449 |
|  |  |  |  |  |  |  |  |  |  |  |
| PM03 | 10.007 | 10.007 | 18.5 | 0.5409 | 17 | 1.0882 | 8.8235 | 2 | 3.3 | 31.2184 |
|  | 10.081 | 10.081 | 19 | 0.5306 | 17.5 | 1.0857 | 8.5714 | 2 | 3.5 | 29.7449 |
|  |  |  |  |  |  |  |  |  |  |  |
| PM04 | 10.029 | 10.029 | 23.5 | 0.4268 | 22 | 1.0682 | 6.8182 | 2 | 3.2 | 32.0053 |
|  | 10.096 | 10.096 | 24 | 0.4207 | 22 | 1.0909 | 9.0909 | 2 | 3.4 | 30.4666 |
|  |  |  |  |  |  |  |  |  |  |  |
| PM05 | 10.07 | 10.07 | 24.5 | 0.4110 | 22 | 1.1136 | 11.3636 | 2 | 3.4 | 31.6075 |
|  | 10.01 | 10.01 | 24 | 0.4171 | 21.5 | 1.1163 | 11.6279 | 2 | 3.3 | 31.2184 |
|  |  |  |  |  |  |  |  |  |  |  |
| PM06 | 10.017 | 10.017 | 21.5 | 0.4659 | 19.5 | 1.1026 | 10.2564 | 2 | 3.5 | 29.7449 |
|  | 10.01 | 10.01 | 22 | 0.455 | 20 | 1.1 | 10 | 2 | 3.4 | 30.4655 |
|  |  |  |  |  |  |  |  |  |  |  |
| PM01A | 10.021 | 10.021 | 21.5 | 0.4661 | 18.4 | 1.1685 | 16.8478 | 2 | 2.9 | 34.59 |
|  | 10.015 | 10.015 | 20 | 0.5008 | 17.5 | 1.1429 | 14.2857 | 2 | 3 | 33.69 |
|  |  |  |  |  |  |  |  |  |  |  |
| PM01B | 10.097 | 18.4 | 0.5488 | 16.2 | 0.6233 | 1.1358 | 13.5802 | 2 | 3.2 | 32.005 |
|  | 10.108 | 19 | 0.532 | 16.4 | 0.6163 | 1.1585 | 15.8537 | 2 | 3.2 | 32.005 |
|  |  |  |  |  |  |  |  |  |  |  |
| PM01C | 10.043 | 21.3 | 0.4715 | 18.98 | 0.5291 | 1.1222 | 12.2234 | 2 | 3.1 | 32.829 |
|  | 10.047 | 21.8 | 0.4609 | 19.1 | 0.5260 | 1.1414 | 14.1361 | 2 | 3.3 | 32.005 |

**S1.2 Appendix. Quality control tests on compressed diclofenac sodium tablets**

S2 Table. Tablet thickness and diameter

| PM01 | | PM02 | | PM03 | | PM04 | | PM05 | |
| --- | --- | --- | --- | --- | --- | --- | --- | --- | --- |
| D | T | D | T | D | T | D | T | D | T |
| 12.01 | 3.01 | 12.08 | 3.04 | 12.07 | 3 | 12.08 | 3.04 | 12.06 | 3.02 |
| 12 | 3 | 12.04 | 3.09 | 12.04 | 3.07 | 12.03 | 3.07 | 12.01 | 3 |
| 12.06 | 3.2 | 12.02 | 3.05 | 12 | 3.04 | 12.01 | 3.02 | 12.04 | 3.07 |
| 12.1 | 3.06 | 12.08 | 3.03 | 12.09 | 3 | 12.02 | 3 | 12.1 | 3.04 |
| 12.08 | 3.08 | 12.1 | 3.07 | 12.1 | 3.02 | 12.1 | 3.09 | 12.06 | 3.13 |
| 12.02 | 3.09 | 12 | 3.04 | 12.02 | 3.04 | 12.04 | 3 | 12.11 | 3.04 |
| 12.01 | 3.01 | 12.04 | 3.04 | 12.07 | 3.04 | 12.03 | 3.02 | 12.04 | 3.02 |
| 12 | 3 | 12.02 | 3.09 | 12.04 | 3 | 12.01 | 3 | 12.1 | 3 |
| 12.06 | 3.2 | 12.08 | 3.05 | 12 | 3.02 | 12.02 | 3.09 | 12.06 | 3.07 |
| 12.1 | 3.06 | 12.1 | 3.03 | 12.09 | 3.04 | 12.1 | 3 | 12.11 | 3.04 |

D is diameter; T is thickness

S3 Table. Tablet thickness and diameter

| PM06 | | PM01A | | PM01B | | PM01C | |
| --- | --- | --- | --- | --- | --- | --- | --- |
| D | T | D | T | D | T | D | T |
| 12.04 | 3.04 | 11.98 | 3.22 | 12.09 | 3.19 | 12.06 | 3.22 |
| 12.07 | 3.02 | 12.06 | 3.17 | 12.15 | 3.12 | 12.42 | 3.16 |
| 12.09 | 3.07 | 11.86 | 3.22 | 11.79 | 3.16 | 11.97 | 3.13 |
| 12.06 | 3 | 12.14 | 3.19 | 12.21 | 3.15 | 12.18 | 3.15 |
| 12.01 | 3.1 | 11.72 | 3.27 | 11.94 | 3.14 | 11.89 | 3.1 |
| 12 | 3.08 | 12.27 | 3.26 | 12 | 3.15 | 11.9 | 3.22 |
| 12.07 | 3.07 | 12.01 | 3.17 | 12.15 | 3.09 | 12.03 | 3.1 |
| 12.09 | 3 | 12.06 | 3.19 | 11.91 | 3.16 | 12.01 | 3.13 |
| 12.06 | 3.1 | 11.72 | 3.22 | 12.09 | 3.14 | 12.1 | 3.09 |
| 12.01 | 3.08 | 11.98 | 3.22 | 12.09 | 3.19 | 12.07 | 3.11 |

D is diameter; T is thickness

S4 Table. Uniformity of weight test

|  | PM01 | PM02 | PM03 | PM04 | PM05 | PM06 | PM01A | PM01B | PM01C |
| --- | --- | --- | --- | --- | --- | --- | --- | --- | --- |
| Average tablet weight per formulation / g | 0.416 | 0.408 | 0.417 | 0.413 | 0.413 | 0.413 | 0.406 | 0.404 | 0.404 |
| 1 | 0.409 | 0.412 | 0.416 | 0.400 | 0.411 | 0.415 | 0.401 | 0.400 | 0.409 |
| 2 | 0.423 | 0.412 | 0.412 | 0.419 | 0.413 | 0.413 | 0.407 | 0.408 | 0.406 |
| 3 | 0.414 | 0.412 | 0.424 | 0.413 | 0.411 | 0.415 | 0.405 | 0.407 | 0.406 |
| 4 | 0.420 | 0.410 | 0.418 | 0.423 | 0.413 | 0.413 | 0.404 | 0.401 | 0.402 |
| 5 | 0.415 | 0.403 | 0.413 | 0.421 | 0.415 | 0.415 | 0.411 | 0.400 | 0.409 |
| 6 | 0.417 | 0.405 | 0.414 | 0.415 | 0.410 | 0.422 | 0.409 | 0.404 | 0.400 |
| 7 | 0.418 | 0.406 | 0.420 | 0.408 | 0.411 | 0.420 | 0.400 | 0.404 | 0.410 |
| 8 | 0.418 | 0.398 | 0.410 | 0.418 | 0.420 | 0.415 | 0.407 | 0.409 | 0.408 |
| 9 | 0.416 | 0.400 | 0.407 | 0.404 | 0.409 | 0.415 | 0.402 | 0.408 | 0.405 |
| 10 | 0.411 | 0.405 | 0.418 | 0.410 | 0.409 | 0.417 | 0.405 | 0.406 | 0.405 |
| 11 | 0.410 | 0.403 | 0.415 | 0.414 | 0.411 | 0.410 | 0.410 | 0.402 | 0.404 |
| 12 | 0.413 | 0.404 | 0.426 | 0.417 | 0.419 | 0.418 | 0.412 | 0.402 | 0.407 |
| 13 | 0.407 | 0.415 | 0.419 | 0.420 | 0.410 | 0.408 | 0.400 | 0.401 | 0.405 |
| 14 | 0.421 | 0.406 | 0.419 | 0.411 | 0.412 | 0.419 | 0.405 | 0.405 | 0.401 |
| 15 | 0.421 | 0.411 | 0.414 | 0.411 | 0.414 | 0.420 | 0.403 | 0.406 | 0.402 |
| 16 | 0.405 | 0.411 | 0.420 | 0.413 | 0.410 | 0.401 | 0.404 | 0.406 | 0.403 |
| 17 | 0.419 | 0.404 | 0.425 | 0.405 | 0.419 | 0.401 | 0.412 | 0.408 | 0.409 |
| 18 | 0.418 | 0.411 | 0.412 | 0.410 | 0.411 | 0.408 | 0.404 | 0.403 | 0.407 |
| 19 | 0.420 | 0.420 | 0.417 | 0.417 | 0.409 | 0.414 | 0.408 | 0.403 | 0.403 |
| 20 | 0.418 | 0.407 | 0.413 | 0.419 | 0.420 | 0.400 | 0.409 | 0.400 | 0.409 |

S5 Table. Tablet hardness /Kgf

| PM01 | PM02 | PM03 | PM04 | PM05 | PM06 | PM01A | PM01B | PM01C |
| --- | --- | --- | --- | --- | --- | --- | --- | --- |
| 7.28 | 6.37 | 7.04 | 8.24 | 7.41 | 8.04 | 8.14 | 8.07 | 9.25 |
| 8.64 | 7.52 | 7.92 | 8.85 | 8.66 | 7.72 | 8.2 | 8.11 | 8.7 |
| 9.02 | 8.47 | 7.54 | 6.79 | 9.29 | 9.52 | 8.69 | 8.29 | 9.16 |
| 8.54 | 7.72 | 7.6 | 8.07 | 8.81 | 9.45 | 9.06 | 8.3 | 8.64 |
| 7.16 | 8.61 | 7.52 | 8.44 | 9.46 | 8.25 | 8.62 | 9.45 | 8.42 |

S6 Table. Tablet friability

| PM01 | PM02 | PM03 | PM04 | PM05 | PM06 | PM01A | PM01B | PM01C |
| --- | --- | --- | --- | --- | --- | --- | --- | --- |
| 0.69 | 0.61 | 0.17 | 0.34 | 0.27 | 0.46 | 0.8 | 0.5 | 0.26 |
| 0.65 | 0.65 | 0.15 | 0.3 | 0.25 | 0.48 | 0.79 | 0.51 | 0.24 |
| 0.63 | 0.57 | 0.19 | 0.38 | 0.29 | 0.44 | 0.78 | 0.49 | 0.26 |

S7 Table. Tensile strength of tablets (MPa)

| PM01 | PM02 | PM03 | PM04 | PM05 | PM06 | PM01A | PM01B | PM01C |
| --- | --- | --- | --- | --- | --- | --- | --- | --- |
| 1.257936 | 1.069525 | 1.203459 | 1.420285 | 1.257294 | 1.372121 | 1.34404 | 1.332775 | 1.501182 |
| 1.499159 | 1.281298 | 1.348282 | 1.521624 | 1.487719 | 1.322938 | 1.366179 | 1.362669 | 1.479044 |
| 1.459976 | 1.445474 | 1.303948 | 1.177178 | 1.598621 | 1.602171 | 1.449371 | 1.417275 | 1.520681 |
| 1.440747 | 1.298166 | 1.309972 | 1.35721 | 1.486369 | 1.631548 | 1.490113 | 1.374524 | 1.493037 |
| 1.202076 | 1.474297 | 1.27807 | 1.452354 | 1.605129 | 1.384158 | 1.424861 | 1.605455 | 1.399618 |

S8 Table. Assay of formulation batches

| Formulation code | Peak area (n=3) | % diclofenac sodium content |
| --- | --- | --- |
| Pure diclofenac powder/ standard | 160.34712 | 100% |
|  |  |  |
| PM01 | 162.10698 | 101.0975 |
|  | 160.109869 | 99.85204 |
|  | 161.00183 | 100.4083 |
|  |  |  |
| PM02 | 167.3544 | 104.3701 |
|  | 158.9754 | 99.14453 |
|  | 160.53832 | 100.1192 |
|  |  |  |
| PM03 | 164.15031 | 102.3718 |
|  | 159.01351 | 99.1683 |
|  | 160.37981 | 100.0204 |
|  |  |  |
| PM04 | 164.9169 | 102.8499 |
|  | 159.9287 | 99.73905 |
|  | 160.1536 | 99.87931 |
|  |  |  |
| PM05 | 166.68091 | 104.7 |
|  | 162.1268 | 101.1099 |
|  | 160.00681 | 99.78777 |
|  |  |  |
| PM06 | 166.88329 | 104.0763 |
|  | 159.98732 | 99.77561 |
|  | 159.97889 | 99.77035 |
|  |  |  |
| PM01A | 160.29416 | 99.96697 |
|  | 160.96241 | 100.3837 |
|  | 160.07532 | 99.83049 |
|  |  |  |
| PM01B | 158.69543 | 98.96993 |
|  | 160.43892 | 100.0573 |
|  | 160.31361 | 99.9791 |
|  |  |  |
| PM01C | 158.00658 | 98.54033 |
|  | 159.88653 | 99.71275 |
|  | 158.88472 | 99.08798 |

**S1.3 Appendix. HPLC method development and validation**

S9 Table. Mean retention time for diclofenac sodium

| Determinations | Retention time / minutes |
| --- | --- |
| 1 | 3.523 |
| 2 | 3.488 |
| 3 | 3.493 |
| 4 | 3.505 |

S10 Table. Mean retention time at 254 nm

| Sample | Average retention time / min (n=4) |
| --- | --- |
| Diclofenac sodium | 3.50 ± 0.016 |

- - 1. S1 Fig. Sample Chromatogram for the testing of the Linearity of the HPLC developed method


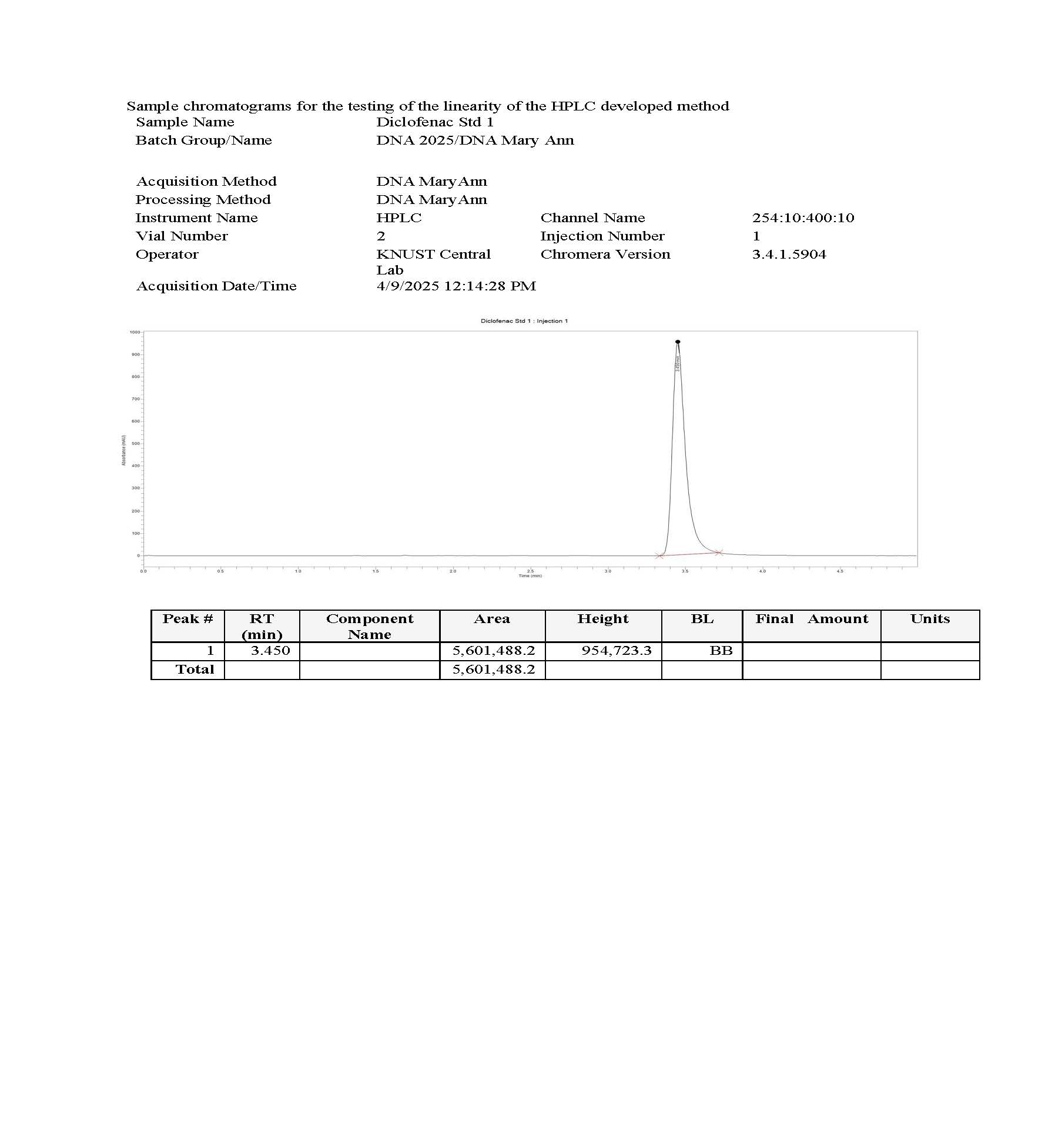


S11 Table. Peak areas for determining linearity

| Concentration of diclofenac sodium in phosphate buffer (pH 6.8) | peak area (n=3) |
| --- | --- |
| 0.03 | 5601488.2 |
|  | 5483541.3 |
|  | 5603254.1 |
|  |  |
| 0.015 | 2678196.3 |
|  | 2708155 |
|  | 2676748.9 |
|  |  |
| 0.0075 | 1229255.4 |
|  | 1225699.9 |
|  | 1218318.2 |
|  |  |
| 0.00375 | 533254.4 |
|  | 534497 |
|  | 537599.4 |
|  |  |
| 0.001875 | 216214.9 |
|  | 212501.7 |
|  | 216174.7 |
|  |  |
| 0.0009375 | 76101.4 |
|  | 73304.3 |
|  | 74777.5 |

S2 Fig. Sample chromatogram for testing the precision of the HPLC developed method


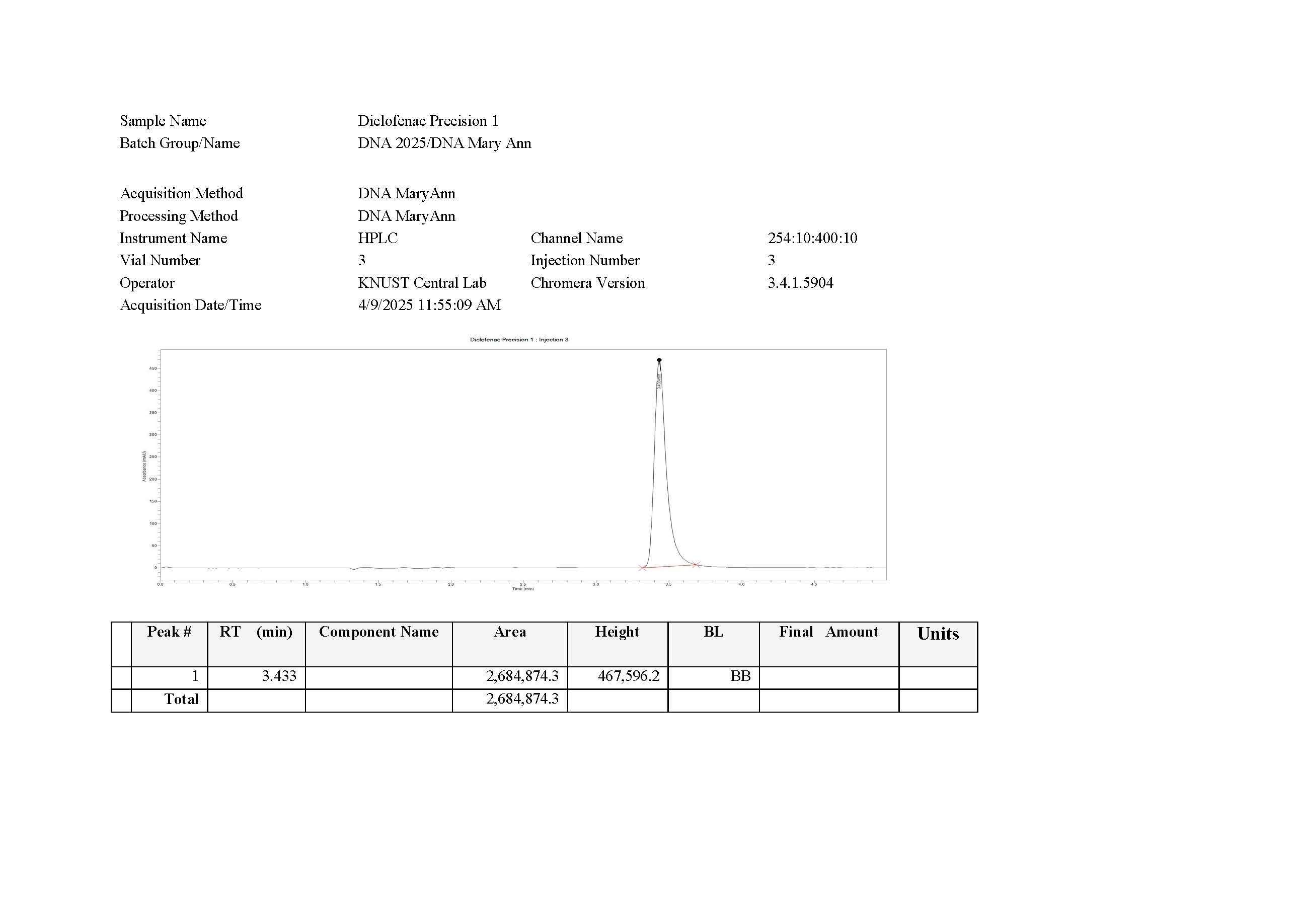


S12 Table. Peak areas for evaluation precision ((% mean recovery and RSD) of the method

| Concentration (µg/ mL) | Determinations | Intra day | | Inter day | | |
| --- | --- | --- | --- | --- | --- | --- |
|  |  | Freshly prepared | After 6 hours | Day 1 | Day 2 | Day 3 |
| 150 | 1 | 2651626.2 | 2662416.2 | 2732638.4 | 2733955 | 2755091.5 |
|  | 2 | 2640954.4 | 2636348.7 | 2745820.2 | 2733955 | 2736348.7 |
|  | 3 | 2662837 | 2655091.5 | 2740337.1 | 2762837.3 | 2749216.7 |
|  | 4 | 2684874.3 | 2636348.7 |  |  |  |
|  | 5 | 2662837.3 | 2649216.7 |  |  |  |
|  | 6 | 2651626.2 |  |  |  |  |

S13 Table. Limit of detection and limit of quantification of diclofenac sodium at wavelength 254 nm

| Sample | **LOD** (%^w^/_v_**)** | **LOQ** (%^w^/_v_**)** |
| --- | --- | --- |
| Diclofenac sodium | 0.00059094 | 0.001790723 |

S14 Table. Peak areas for evaluation of accuracy ((% mean recovery and RSD) of the method

| Determinations of peak areas | Concentration of diclofenac sodium in phosphate buffer (pH 6.8) /%^w^/_v_ | | |
| --- | --- | --- | --- |
| 1 | 0.03 | 0.015 | 0.0075 |
| 2 | 5601488.2 | 2678196.3 | 1229255.4 |
| 3 | 5483541.3 | 2708155 | 1225699.9 |

S3 Fig. Sample peak for accuracy of HPLC developed method


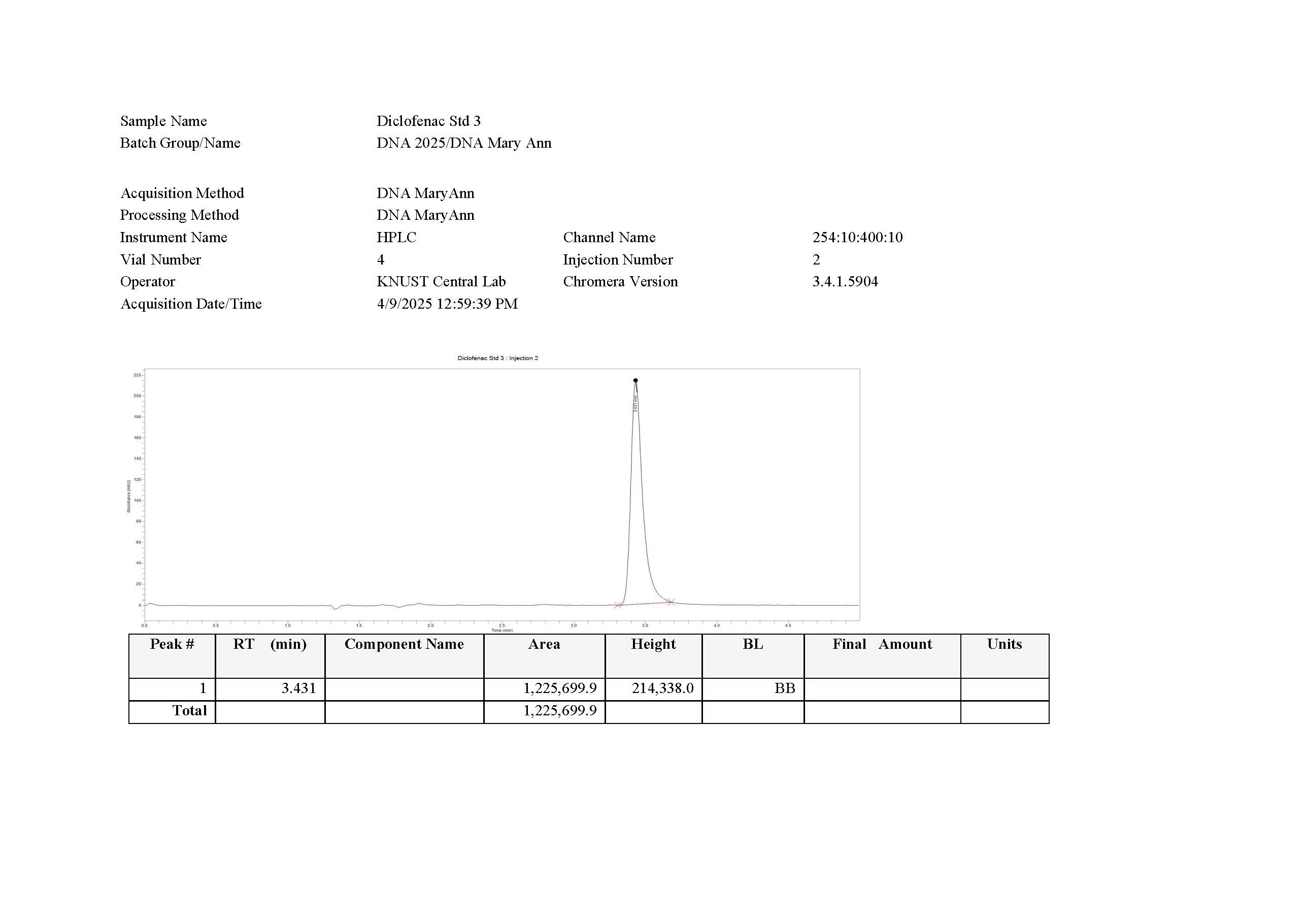


S4. Fig. Sample chromatogram for the determination of linear regression for robustness of the HPLC developed method


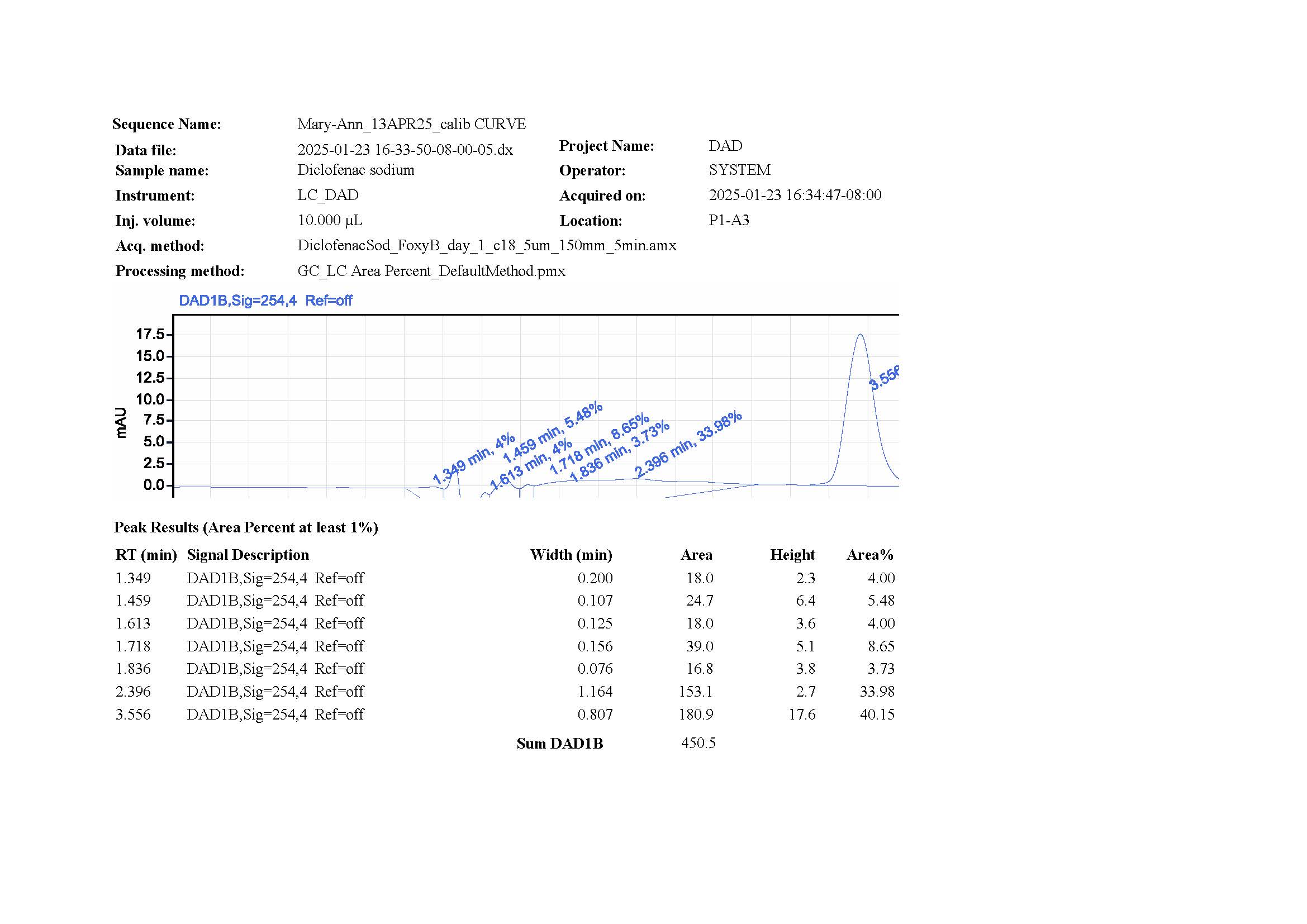


S15 Table. Peaks for calibration curve for determining the equation of the straight line in evaluating the robustness of the HPLC developed method

| Concentration of diclofenac sodium in phosphate buffer (pH 6.8) /%^w^/_v_ | peak area | |
| --- | --- | --- |
|  | 1 | 2 |
| 0.03 | 2856 | 2853.5 |
| 0.015 | 1433.8 | 1439 |
| 0.0075 | 726.5 | 726.7 |
| 0.00375 | 365.2 | 364.4 |
| 0.001875 | 180.9 | 180.8 |
| 0.0009375 | 87.6 | 87.4 |

S5 Fig. Calibration curve for evaluating the robustness of the method

S16 Table. Peak areas for evaluation of the robustness (% mean recovery and RSD) of the method

| Determinations of peak areas | Concentration of diclofenac sodium in phosphate buffer (pH 6.8) /%^w^/_v_ | | |
| --- | --- | --- | --- |
| 1 | 0.03 | 0.015 | 0.0075 |
| 2 | 2856 | 1433.8 | 726.5 |
| 3 | 2853.5 | 1439 | 726.7 |
| 4 | 2854 | 1436 | 725.8 |

S6 Fig. Sample chromatogram for diclofenac release studies at 6 hours (PM01)


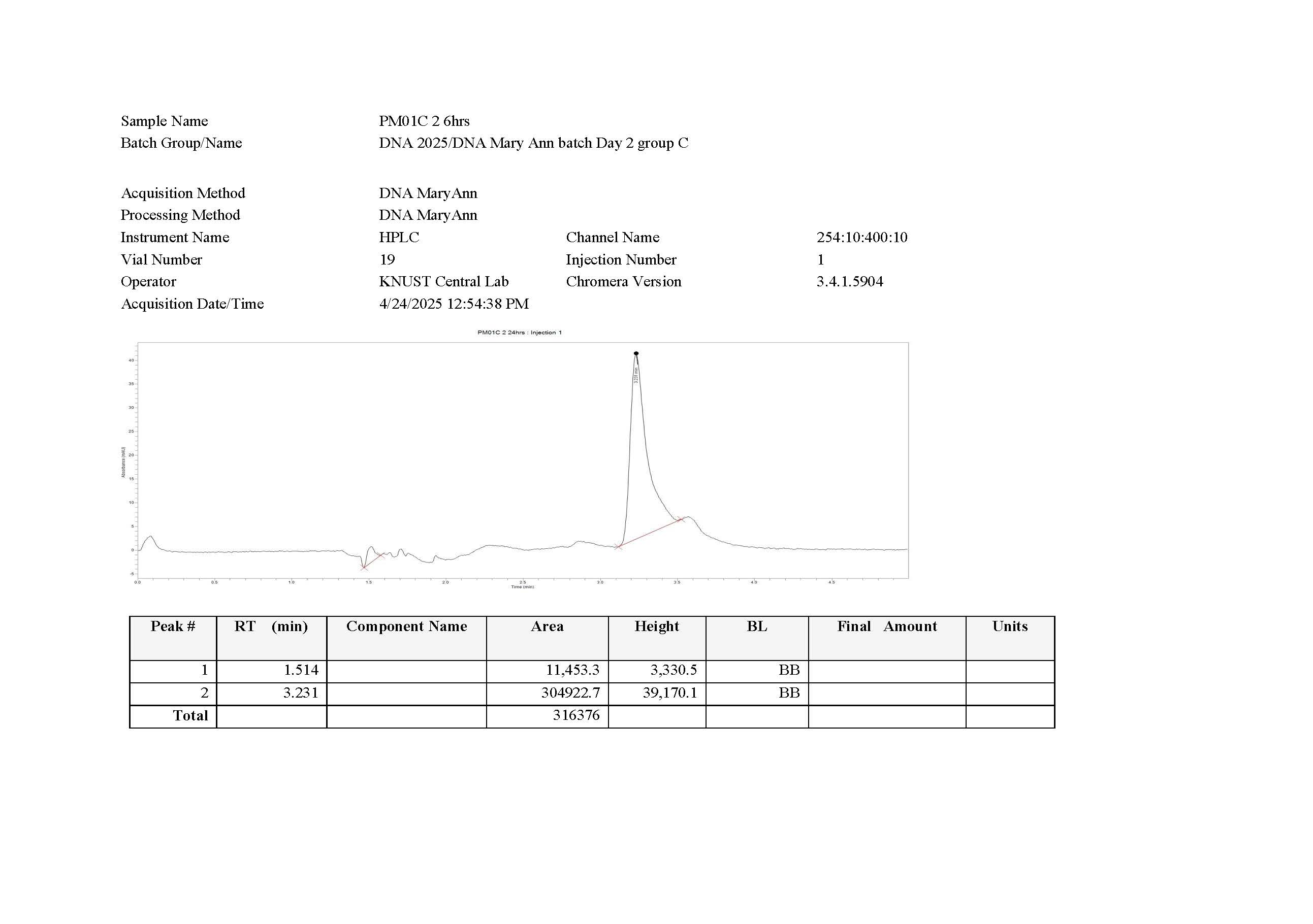


S17 Table. Cumulative diclofenac sodium release from formulation batches PM06

| Time/h | Average Peak area (n = 6) | % Cumulative drug release |
| --- | --- | --- |
| 0.083 | 10442.975 ± 1128.724 | 7.77 ± 0.055 |
| 0.25 | 285933.450 ± 3688.956 | 20.91 ± 0.173 |
| 0.5 | 821992.225 ± 20172.589 | 46.53 ± 0.954 |
| 0.75 | 1105079.600 ± 4217.428 | 60.46 ± 0.199 |
| 1 | 1461614.375 ± 20625.078 | 78.01 ± 0.985 |
| 1.25 | 1674092.225 ± 15539.328 | 100.355 ± 0.839 |

S18 Table. Cumulative diclofenac sodium release from formulation batches PM01 – PM05

| Time /h | PM01 | | PM02 | | PM03 | | PM04 | | PM05 | |
| --- | --- | --- | --- | --- | --- | --- | --- | --- | --- | --- |
|  | Mean peak area | %CM | Mean peak area | %CM | Mean peak area | %CM | Mean peak area | %CM | Mean peak area | %CM |
| 0.75 | 971299.32 ± 18198.56 | 53.29 ± 0.86 | 1230567.28 ± 3241.2 | 65.57 ± 0.16 | 1466773.93 ± 26780.26 | 70.21 ± 1.27 | 1454090.03 ± 12031.23 | 76.16 ± 0.57 | 1664852.25 ± 21473.08 | 86.14 ± 1.02 |
| 2 | 1555199.45 ± 21171.28 | 81.54 ± 1.014 | 1711150.85 ± 17915.76 | 89.06 ± 0.85 | 1926105.48 ± 36978.10 | 92.75 ± 1.75 | 1839957.83 ± 20063.83 | 95.28 ± 0.95 | 1790173.35 ± 7584.82 | 93.03 ± 0.37 |
| 6 | 1779117.37 ± 17277.60 | 93.05 ± 0.84 | 1808256.97 ± 4812.112 | 94.65 ± 0.22 | 2041448.45 ± 36063.96 | 99.23 ± 1.70 | 1892644.52 ± 21572.92 | 98.83 ± 1.01 | 1852999.63 ± 24789.87 | 97.04 ± 1.16 |
| 10 | 1906977 ± 39671.55 | 100.12 ± 1.89 | 1879684.22 ± 39946.01 | 99.06 ± 1.90 | 1991096.42 ± 6633.18 | 97.93 ± 0.31 | 1828193.52 ± 24166.35 | 96.85 ± 1.15 | 1875366.35 ± 15889.82 | 99.15 ± 0.76 |
| 12 | 1887812.3 ± 27706.80 | 100.30 ± 1.352 | 1896479.18 ± 21042.30 | 100.93 ± 1.013 | 1942847.28 ± 29031.84 | 96.70 ± 1.39 | 1778496.38 ± 18823.61 | 95.54 ± 0.89 | 1916616.25 ± 2635.74 | 102.17 ± 0.12 |

%CM – Percentage cumulative diclofenac sodium release

S19 Table. Peak areas for formulation batches PM01A - PM01C and ST

| Time /h | PM01A | | PM01B | | PM01C | | ST | |
| --- | --- | --- | --- | --- | --- | --- | --- | --- |
|  | Mean peak area | %CM | Mean peak area | %CM | Mean peak area | %CM | Mean peak area | %CM |
| 0.75 | 771859.78 ± 6900.85 | 43.84 ± 0.33 | 693710.10 ± 10060.12 | 40.14 ± 0.48 | 18384.73 ± 1882.66 | 8.15 ± 0.09 |  |  |
| 2 | 1362860.68 ± 3535.06 | 72.32 ± 0.17 | 1290986.93 ± 42070.85 | 68.88 ± 2.00 | 264593.13 ± 10745.43 | 19.90 ± 0.51 | 276259.23 ± 14476.87 | 20.36 ± 0.69 |
| 6 | 1560933.05 ± 7696.75 | 82.50 ± 0.37 | 1503764.75 ± 50042.66 | 79.72 ± 2.26 | 317581.1 ± 20085.98 | 22.63 ± 0.96 | 433976.58 ± 18363.27 | 28.06 ± 0.86 |
| 10 | 1759998.03 ± 16072.81 | 92.84 ± 0.75 | 1595291.98 ± 10340.39 | 84.92 ± 0.49 | 904122.48 ± 18467.68 | 50.02 ± 0.51 | 915970.45 ± 21623.30 | 51.20 ± 1.03 |
| 12 | 1863828.50 ± 41190.53 | 98.77 ± 1.95 | 1725712.33 ± 12104.90 | 92.02 ± 0.56 | 1026398.55 ± 11224.08 | 57.02 ± 0.52 | 1037123.55 ± 35275.53 | 57.50 ± 1.69 |
| 16 | 1874895.15 ± 29082.19 | 100.35 ± 1.39 | 1834525.58 ± 19724.01 | 98.17 ± 0.94 | 1242613.28 ± 45667.04 | 67.88 ± 2.18 | 1220382.88 ± 9458.05 | 66.81 ± 0.42 |
| 18 | 1817661.90 ± 20979.29 | 98.71 ± 1.01 | 1840983.13 ± 42141.33 | 99.52 ± 1.98 | 1395470.15 ± 1595.30 | 75.85 ± 0.10 | 1433405.48 ± 21801.41 | 77.62 ± 1.06 |
| 21 | 1768956.05 ± 22724.33 | 97.44 ± 1.11 | 1843685.30 ± 14067.89 | 100.70 ± 0.69 | 1561259.00 ± 5815.04 | 84.52 ± 0.31 | 1536952.15 ± 18221.82 | 83.36 ± 0.90 |
| 24 | 1719982.70 ± 6248.06 | 97.19 ± 0.33 | 1791542.38 ± 44481.39 | 99.28 ± 2.08 | 1874455.98 ± 45470.11 | 100.26 ± 2.12 | 1846738.45 ± 5711.21 | 98.94 ± 0.28 |

%CM – Percentage cumulative diclofenac sodium release

## **S1.4 Appendix. Release from matrix formulations**

S7 Fig. Graph for zero order kinetics for diclofenac sodium release from matrix tablets

S20 Table. Equation of the linear regression for zero order kinetics of diclofenac sodium release from matrix tablets

| Formulation code | Equation of the straight line | R^2^ |
| --- | --- | --- |
| PM01A | y = 1.818x + 64.94 | 0.6396 |
| PM01B | y = 2.118x + 58.99 | 0.7617 |
| PM01C | y = 3.807x + 7.674 | 0.9817 |
| ST | y = 3.561x + 11.96 | 0.9850 |

S8 Fig. Graph for first order kinetics for diclofenac sodium release from matrix tablets

S21 Table. Equation of the linear regression for first order kinetics of diclofenac sodium release from matrix tablets

| Formulation code | Equation of the straight line | R^2^ |
| --- | --- | --- |
| PM01A | y = -0.06728x + 1.543 | 0.6509 |
| PM01B | y = -0.08375x + 1.785 | 0.8021 |
| PM01C | y = -0.04140x + 2.069 | 0.8483 |
| ST | y = -0.06656x + 2.304 | 0.6815 |

S9 Fig. Graph for Higuchi model of diclofenac sodium release from matrix tablets

S22 Table. Equation of the linear regression for Higuchi model of diclofenac sodium release from matrix tablets

| Formulation code | Equation of the straight line | R^2^ |
| --- | --- | --- |
| PM01A | y = 11.90x + 48.67 | 0.7964 |
| PM01B | y = 13.37x + 41.63 | 0.8816 |
| PM01C | y = 21.98x - 16.93 | 0.9512 |
| ST | y = 22.69x - 19.55 | 0.9591 |

S10 Fig. Graph for Hixson-Crowell model for diclofenac sodium release from matrix tablets

S23 Table. Equation of the linear regression for HC model of diclofenac sodium release from matrix tablets

| Formulation code | Equation of the straight line | R^2^ |
| --- | --- | --- |
| PM01A | y = -0.1100x + 3.094 | 0.4807 |
| PM01B | y = -0.1670x + 3.803 | 0.7667 |
| PM01C | y = -0.1570x + 5.065 | 0.6855 |
| ST | y = -0.1297x + 4.917 | 0.8580 |

S11 Fig. Graph for Korsemeyer Peppas model for diclofenac sodium release from matrix tablets

S24 Table. Equation of the linear regression for Korsemeyer Peppas model of diclofenac sodium release from matrix tablets

| Formulation code | Equation of the straight line | R^2^ |
| --- | --- | --- |
| PM01A | y = 0.2159x + 1.731 | 0.8878 |
| PM01B | y = 0.2421x + 1.694 | 0.9196 |
| PM01C | y = 0.6941x + 0.9973 | 0.9570 |
| ST | y = 0.6559x + 1.050 | 0.9524 |
